# A Simple Method for Simulating Groundwater Interactions with Fens to Forecast Development Effects

**DOI:** 10.1111/gwat.12931

**Published:** 2019-08-20

**Authors:** Daniel T. Feinstein, David J. Hart, Sarah Gatzke, Randall J. Hunt, Richard G. Niswonger, Michael N. Fienen

**Affiliations:** ^1^ U.S. Geological Survey, Upper Midwest Water Science Center, University of Wisconsin‐Milwaukee, Lapham Hall, Room 338, 3209 North Maryland Avenue Milwaukee WI 53211 Survey; ^2^ The Nature Conservancy, Mukwonago River Watershed Office East Troy WI 53120–1836; ^3^ U.S. Geological Survey, Upper Midwest Water Science Center, 8505 Research Way Middleton WI 53562; ^4^ U.S. Geological Survey, Water Resources Mission Area, 345 Middlefield Road Menlo Park CA 94025

## Abstract

Protection of fens–wetlands dependent on groundwater discharge–requires characterization of groundwater sources and stresses. Because instrumentation and numerical modeling of fens is labor intensive, easy‐to‐apply methods that model fen distribution and their vulnerability to development are desirable. Here we demonstrate that fen areas can be simulated using existing steady‐state MODFLOW models when the unsaturated zone flow (UZF) package is included. In cells where the water table is near land surface, the UZF package calculates a head difference and scaled conductance at these “seepage drain” cells to generate average rates of vertical seepage to the land. This formulation, which represents an alternative to blanketing the MODFLOW domain with drains, requires very little input from the user because unsaturated flow‐routing is inactive and results are primarily driven by easily obtained topographic information. Like the drain approach, it has the advantage that the distribution of seepage areas is not predetermined by the modeler, but rather emerges from simulated heads. Beyond the drain approach, it takes account of intracell land surface variation to explicitly quantify multiple surficial flows corresponding to infiltration, rejected recharge, recharge and land‐surface seepage. Application of the method to a basin in southeastern Wisconsin demonstrates how it can be used as a decision‐support tool to first, reproduce fen distribution and, second, forecast drawdown and reduced seepage at fens in response to shallow pumping.

## Abstract 2

*Article impact statement*: Protection of fens is improved by enhanced fen‐groundwater exchange simulation provided by this easy‐to‐apply approach.

## Introduction and Background

There is wide interest in protecting groundwater‐fed wetlands by developing tools to assess effects of pumping and land‐use changes on near‐surface hydrologic conditions (Shedlock et al. 1993; Hunt et al. 1996; Batelaan et al. 2003; Whittington and Price 2006; Poff et al. 2009; Huntington and Niswonger 2012; Rossi et al. 2012; Aldous and Bach 2014; Leaf et al. 2015; Sampath et al. 2016). Methods applied for modeling groundwater exchange with wetlands include blanketing a MODFLOW model with drains that become active when the water table elevation is at the land surface (e.g., Halford 1997) and using digital elevation models in conjunction with simulations of water‐table depth to delineate areas of exchange (e.g., Rossman et al. 2019).

This paper presents a method for delineating fen locations and seepage that is easily incorporated into existing regional MODFLOW models by addition of the unsaturated zone flow (UZF) package for MODFLOW (Niswonger et al. 2006). Although the UZF package is ordinarily applied to simulate unsaturated flow above the water table, this approach takes advantage of UZF functionality for simulating saturation excess and spring discharge; thus, the application does not require specification of the aquifer's unsaturated zone properties—only the saturated hydraulic conductivity for the near‐surface region is required.

**Figure 1:**
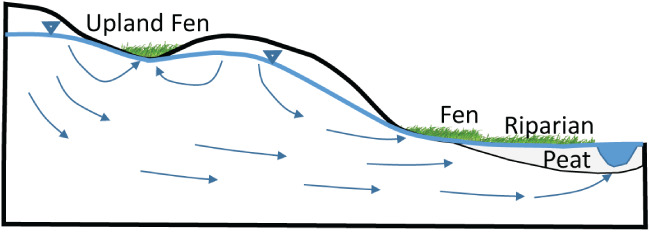
Relation of fens to landscape.

The method is well suited to simulate fens, defined here as wetlands dependent on groundwater discharge. Fens can occur on slopes, flats or depressions where groundwater levels are near or above the land surface creating alkaline water chemistry in the wetland (Figure 1). However, the approach described here is applicable to any groundwater dependent surface water feature (e.g., springs).

Groundwater discharge to the land surface occupied by fens is defined as “seepage,” while “infiltration” is defined as the portion of precipitation that crosses the root zone and percolates to the top of the unsaturated zone. The part of infiltration that reaches the water table is called “recharge,” the part that is rejected when the water table rises to near the land surface is called “rejected recharge” due to saturation excess. Fens are often found in low‐lying areas in temperate watersheds (e.g., riparian corridors of streams), but can be found anywhere in a watershed where groundwater discharge to the land surface is relatively stable (Aldous and Bach 2014; Sampath et al. 2016). Moreover, fens are associated with locations where the water‐table elevations are not only relatively stable but also shallow, typically within a foot (less than a meter) of the land surface. The shallow or even ponded water tables produce wet ground conditions, which in turn support assemblages of hydrophytic vegetation. The relatively stable hydrologic conditions associated with fens means that steady‐state assumptions, those that neglect transient and seasonal conditions, are often sufficiently representative for management purposes.

**Figure 2:**
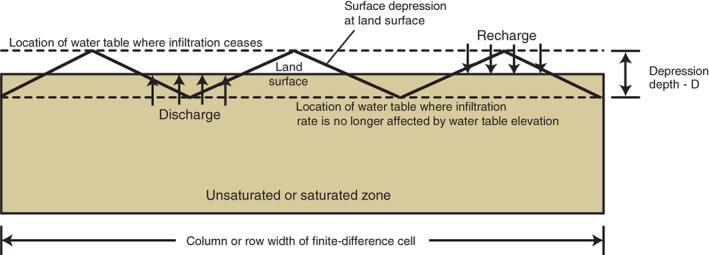
The effect of surface depression depth, D, at land surface on groundwater recharge and discharge in relation to the water table in a MODFLOW cell.

In groundwater modeling, it is customary to simulate fens and related features such as springs and seeps by imposing head‐dependent boundary conditions (Anderson et al. 2015). These conditions (such as the DRN package in MODFLOW) are constructed by the modeler as an input rather than simulated as an output. The alternative method presented here allows a MODFLOW solution to determine the location of fens and their seepage rates. In more general terms, rather than fixing the location of recharge and discharge areas through MOFLOW input, this method simulates dynamic recharge and discharge conditions as a function of groundwater head. The capability to convert from recharge to discharge conditions as a function of the head solution is an advantage from the perspective of removing model bias and increasing the power of the calibration process to reveal controls on flow by matching simulated seepage to mapped fen locations. The method allows the amount of seepage to be drawn directly from model inputs (most importantly, from topography) that in some cases are better known than boundary condition properties such as drain conductances. Finally, the approach uses the concept of topographic “surface depression depth” of a model cell to calculate “subgridded” flows that correspond to seepage, rejected recharge and recharge (Figure 2), all potentially active in the same MODFLOW cell at the same time. These latter capabilities can be important for simulating lateral flows among fen complexes that are connected through shallow groundwater.

The proposed method is easy to apply because it requires very little change to existing regional or subregional MODFLOW flow models. An example is presented where forecasts of pumping effects are simulated for fens in a southeastern Wisconsin basin.

## Representing Fens Using the UZF Package

The gist of the method is to add the capacity to simulate seepage to fens and other groundwater‐dependent ecosystems within a MODFLOW model. This increased capability is added without the introduction of new boundary conditions and without explicit simulation of unsaturated or transient flow conditions. The MODFLOW UZF package (Niswonger and Prudic 2004; Niswonger et al. 2006; Niswonger et al. 2011) inserts a dynamic condition at water‐table cells which allows seepage discharge to the land surface and reduced recharge from infiltration whenever the simulated water table is above a specified depth below the land surface. These seepage locations are not specified beforehand by the user—instead they automatically become active as a function of groundwater head. That is, seepage to the land surface arises automatically as a model output from the specification of the model geometry, boundary conditions, infiltration rates, and aquifer properties.

Accurate land surface elevation is a crucial input for fen simulation because seepage occurrence and magnitude are calculated using groundwater head for each cell. Groundwater head in the cell does not have to equal the land surface elevation for a seepage to occur. Rather, the UZF package variable *SURFDEP* (Niswonger 2009) allows the user to input a surface depression depth, *D*, to account for the topographic variation of the land surface within a model cell (Figure 2). Seepage is calculated according to Darcy's Law:

(1) $$Q_{\text{seepage}}=\frac{A\left(h\right)K_{v}\partial h}{\partial z},$$

and

(2) $$A\left(h\right)=A_{\text{cell}}\left(h_{\mathrm{gw}}-z_{\text{bfen}}\right)/D.$$

where *Q*_seepage_ is the groundwater seepage to fens; *K*_*v*_ is vertical hydraulic conductivity corresponding either to the aquifer *K*_*v*_ or to a special UZF array for the saturated *K*_*v*_ of the unsaturated zone; *h*_gw_ is groundwater head or water table altitude; *A*(*h*) is the plan‐view area of surface depressions below *h*_gw_ and must be greater than zero, and *A*_cell_ is the plan‐view area of the cell; and *D* is the range of surface depression depth within *A*_cell_ that is typically calculated by resampling a digital elevation model to the model grid cells;. Fen bottom elevation (*z*_bfen_) is not explicitly simulated in this simplified approach, and it is assumed that the fen bottom elevation can be approximated by the lowest altitude of surface depressions in the cell (the zero‐depth approximation presented in Wallach et al. 1997):

(3) $$z_{\text{bfen}}=\mathrm{TOP}1-\frac{D}{2},$$

and the vertical hydraulic gradient is approximated as:

(4) $$\partial h/\partial z=\frac{(h_{\mathrm{gw}}-\left(\mathrm{TOP}1-D/2\right)}{b/2},$$

where *b* is the cell thickness in the vertical direction; *h* is head; *TOP*1 is the cell top attitude. Seepage (or “surface leakage”) is nonzero whenever the simulated water table is above an elevation equal to *TOP*1 *− D*/2.

**Figure 3:**
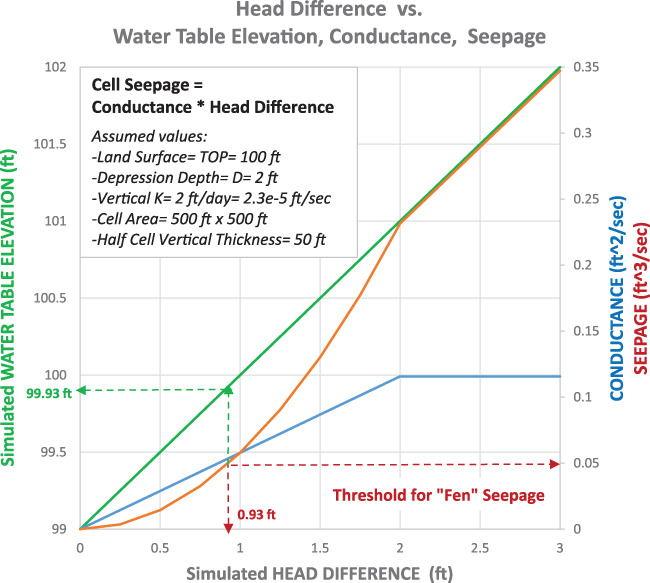
Example calculation of seepage flux as function of water table elevation and corresponding water table elevation for fen threshold.

The seepage condition expression can be reformulated as the product of an unconfined conductance term, equal to (A[h]*K_*v*_ *)/(b/2*), and a head difference, equal to (*h*_gw_ *–z*_bfen_), where *z*_bfen_ is a constant for the cell, equal to *TOP*1 − *D*/2. The conductance term can be interpreted as a positive function of the fraction of undulating land surface inside the cell that is below the simulated water table elevation. It rises linearly with the rise in water table elevation until it becomes constant at the elevation *TOP*1 + *D*/2. The seepage discharge term is parabolically increasing from *TOP*1 − *D*/2 to *TOP*1 + *D*/2, and then linearly increasing for higher water table solutions (Figure 3).

**Figure 4:**
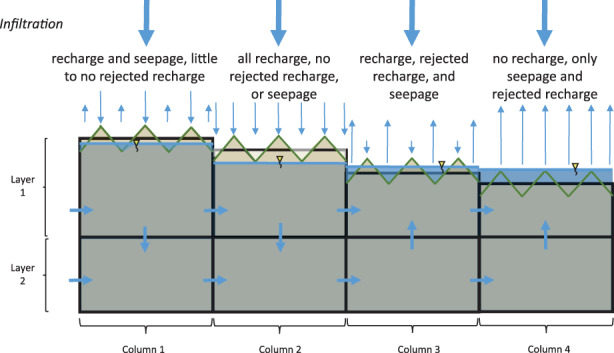
Subgridded flows for different relations of water table elevation to the surface depression depth.

In application, the UZF solution uses surface depression depth to track the water budget of three potentially co‐existing flows: rejected recharge, seepage, and recharge. Rejected recharge, associated with Dunnian runoff, is zero when the water table elevation is below *TOP*1 − *D*/2 and increases linearly to 100% of applied infiltration when the water table elevation is at or above *TOP1* + *D*/2. Recharge (water that that arrives at the water table) is calculated as the difference between infiltration and rejected recharge. When the surface depression depth is greater than zero, seepage can co‐exist with rejected recharge and UZF recharge in the same cell (Table 1). In this sense the solution is subgridded: rejected recharge and seepage are associated with parts of the cell where there are surface depressions, recharge in areas of the cell outside the surface depressions (Figure 1, and also Figure 4, which is a simplification of Figure 1 from the discretized model perspective). Each of these flows is calculated without consideration of the unsaturated zone or temporal lags—they are controlled instead by the solved water‐table elevation in an iterative and nonlinear way that reflects the change in UZF recharge and seepage that occurs as a function of groundwater head. It is worth emphasizing that the ability of the simple UZF method to effectively insert “seepage drains” where needed, compute an appropriate conductance on the basis of existing model inputs, and simulate subgridded inflows and outflow as a function of the simulated water‐table solution, are advantages that are not realized if MODFLOW drain boundary conditions are simply specified at locations that the user preselects as cells with potential for seepage.

**Table 1:** Example Subgrid Flows for Cells with and Without Seepage

Variable	Unit	Large Seep	Small Seep
TOP elevation of Layer 1	ft	840.11	809.73
BOTTOM elevation of Layer 1	ft	740.11	709.73
Thickness of Layer 1	ft	100.00	100.00
Simulated head in Layer 1	ft	842.466	808.860
Simulated head in Layer 2	ft	844.920	809.01
Head relative to land surface	ft	2.36	−0.87
INFILTRATION	ft^3^/s	0.0055	0.0053
RECHARGE	ft^3^/s	0.0000	0.0050
REJECTED RECHARGE	ft^3^/s	0.0055	0.0003
SEEPAGE	ft^3^/s	0.3260	0.0010
GROUNDWATER RUNOFF	ft^3^/s	0.3315	0.0013
= rejected recharge + seepage			
*Simulated water table's*		*Water table more than*	*Water table less than*
*relation to land surface*		1 ft (>0.5*D) above TOP	1 ft (<0.5*D) below TOP
		*=> above surface depression rise*	=> *within surface depression depth*
*Subgrid flows*		*All INFILTRATION is REJECTED*	*6.5% of INFILTRATION is REJECTED*
		RECHARGE = 0	*93.5% INFILTRATION to RECHARGE*
		*SEEPAGE > 0.05 ft* ^*3*^ */s*	*SEEPAGE < 0.05 ft* ^*3*^ */s*
		*= > FEN cell*	*= > not FEN cell*

From a groundwater perspective, fens can be defined in terms of a threshold amount of seepage, that is, a flux assumed to be high enough over the cell area to support the plant life associated with fens. For the assumed parameter values in Figure 3 (typical for the example southeast Wisconsin basin), the choice of a fen threshold equal to 0.05 ft^3^/s (0.0014 m^3^/s) results in fen generation at a water table elevation no deeper than 0.07 foot (0.02 m) below land surface. Note that the *D*/2 depth was estimated to be 1 foot (0.3 m) below land surface: seepage generation begins at that depth, but fen generation for the assumed threshold only occurs when the water table rises almost to the land surface elevation.

In some ways the UZF method for simulating seepage presented here is similar to the older method of blanketing all model cells in layer 1 with MODFLOW drains and allowing the solution to dictate which cells are active seepage sites. It does this by determining where the simulated water table is above the land surface, equated with the drain level set by the user (e.g., see Halford 1997). However, the UZF method differs from, and offers potential improvements to, the blanketed drain method in the following ways:

1. The presence of seepage is determined by the relation of the simulated water table to the bottom depth of surface depressions below the land surface;
2. The amount of seepage is dictated not only by the relation of the simulated water table to the amplitude of the assumed undulations in the land‐surface, but also by a conductance term which is partly a function of a UZF parameter array which corresponds to the surficial (or soil) vertical hydraulic conductivity;
3. The same formulation used within the UZF package to calculate seepage on a cell‐by‐cell basis is also used to partition the infiltration input by the user between recharge that arrives at the water table and rejected recharge which runs off the land surface;
4. All four surficial flows—infiltration (an input array) and seepage, recharge and rejected recharge (output arrays) are calculated and budgeted on a cell‐by cell basis;
5. Seepage and rejected recharge can be routed to streams and lakes via the MODFLOW SFR and LAK packages.

## Overview of Example Groundwater Flow Model

**Figure 5:**
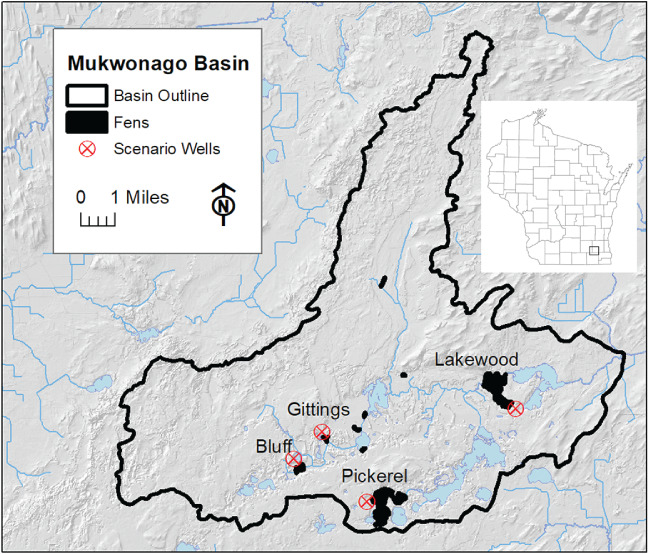
Location map for Mukwonago Basin with named fens. Terrain data from Wisconsin View website, https://www.wisconsinview.org/.

The outlined method of delineating fen distribution and discharge with the MODFLOW UZF package was applied to an inset model extracted from the northern (Wisconsin) half of a published USGS steady‐state model of the Upper Fox River basin in the U.S. Upper Midwest (Feinstein et al. 2018). The extracted model incorporates the Mukwonago River basin, a 10‐digit hydrologic unit code (HUC10) basin occupying 86.2 mi^2^ (223 km^2^) in southeastern Wisconsin (Figure 5). Portions of the headwaters of the Mukwonago Basin are designated as “outstanding and exceptional resource waters” by the Wisconsin State Legislature. This basin is largely agricultural (46% of area), rich in wetlands (11% of area), and relatively less developed in terms of groundwater use than surrounding areas (Southeastern Wisconsin Regional Planning Commission 2010; Wisconsin Department of Natural Resources 2019). The model input is documented in both the online Supporting Information and in the U.S. Geological Survey model archive (Feinstein et al. 2019) accompanying this article. Worth noting here are the following features of the model:

- The model hydrogeology consists of a glacial aquifer overlying sedimentary bedrock aquifers;
- The extracted steady‐state model simulates both regional and local groundwater components of flow, accommodating long flow paths (typically, involving the bedrock units and discharge across basins or to deep wells), and short flow paths (discharging predominantly through glacial material to nearby surface water, wetland features, or shallow wells);
- The extraction preserves many elements of the parent 15‐layer Upper Fox model (Jones and Feinstein 2018), including the vertical layering dedicated to the shallow glacial part of the flow system and the layering dedicated to the deeper bedrock units, as well as a uniform 500 × 500 ft^2^ (152.4 × 152.4 m^2^) lateral discretization for the entire model domain;
- The glacial material in the Mukwonago Basin portion of the extracted model averages 150 feet (46 m) thick, varying between 0 and about 300 ft (92 m) thick, and is represented by the top one, two, or three model layers, depending on total glacial thickness;
- The hydraulic conductivity distribution of glacial deposits within the Mukwonago Basin, which consists largely of sandy material but with appreciable pockets of silt and clay, has been updated to incorporate more driller log information at a finer scale than was used in the parent model;
- The infiltration is inherited from the parent model (Westenbroek et al. 2009; Feinstein et al. 2018); within the Mukwonago Basin it totals 44.58 ft^3^/s (1.263 m^3^/s) equivalent to an average rate of 7.6 in/year (0.19 m/year). By way of comparison the estimated and simulated steam baseflow at the outlet of the Mukwonago Basin is 39 ft^3^/s (1.10 m^3^/s)—see online Supporting Information for more details.
- The surface water network in the Mukwonago study area (Figure 5) consists of streams (the Mukwonago River and its tributaries), of lakes (which are largely integrated with the stream network), and of springs and wetlands (including riparian features along the streams and somewhat more upland fens, both fed by groundwater seepage).
- In MODFLOW, the streams are represented by the SFR2 (streamflow routing) package, the lakes by the DRN (drain) package, and the riparian and fen features by the UZF package;
- The riparian corridor for the example model is defined by the 500 feet (152.4 m) grid spacing; UZF seepage and rejected recharge occurring within a SFR cell is considered to behave as direct discharge to the channel, whereas UZF seepage occurring outside of cells hosting streams is equated with fen discharge if the cell flux surpasses the fen threshold of to 0.05 ft^3^/s (0.0014 m^3^/s);
- The surface depression depth D, equal to 2 feet (0.61 m) for the basin was calculated from the standard deviation of the range of LIDAR land surface elevations in model cells associated with zones mapped by the Southeastern Wisconsin Planning Commission as containing wet soils or standing water over at least a quarter of the cell area (Wisconsin Department of Natural Resources 2011);
- The calibration process applied to the extracted model yielded good agreement between simulated head and flux values and (1) Mukwonago Basin water‐level measurements, (2) estimated baseflow at the Mukwonago river basin outlet, and (3) measured baseflow at stream gauge locations within the basin. The horizontal and vertical conductivity of the glacial material as well as the properties of the stream and lake beds and a multiplier on infiltration were updated as result of the calibration process.

Please refer to the online Supporting Information for more details on model construction and calibration, including preparation of the steady‐state UZF file central to the proposed method. This file contains (1) an entry for the surface depression depth, (2) an array showing where in what water‐table cells UZF is active, (3) an array of infiltration rates, and, optionally, (4) an array to route seepage and rejected recharge (groundwater runoff) to the nearest topographically‐downgradient stream or lake. This last input is only needed if the user wants to add groundwater runoff to baseflow simulated as direct channel discharge (useful for calibration). Note that parameters controlling unsaturated flow (such as residual and saturated water content, wilting point and Brook‐Corey exponent) are unneeded and omitted from the model UZF file. The user can turn off the unsaturated calculations with a flag in the header line of the UZF file.

## Example Results

**Figure 6:**
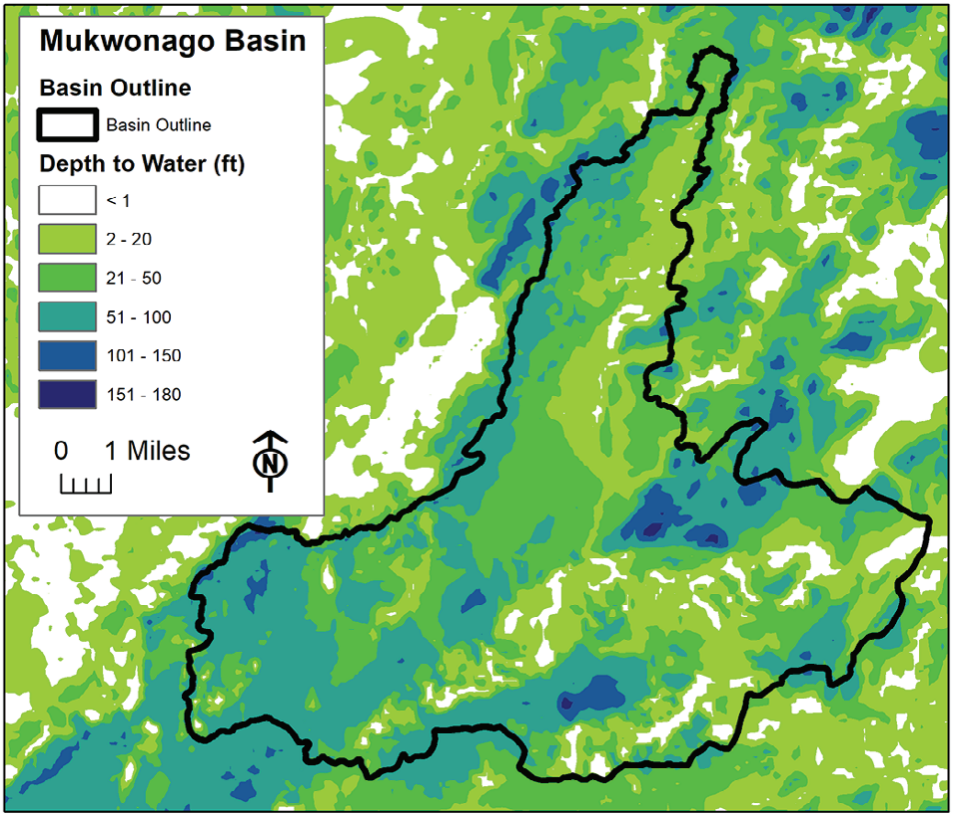
Simulated depth to water table in vicinity of Mukwonago Basin.

The subgridded flow accounting included in the UZF package provides a more detailed water budget than is typically output by MODFLOW. For the input described above, the simulated budget for the Mukwonago Basin portion of the extracted model (Table 2) indicates that about 2.5% of the total applied infiltration is rejected due to high water table. The total seepage flux—17.88 ft^3^/d (0.5063 m^3^/d)—occurs over 5.3% of the basin cells. They correspond to the white areas in Figure 6, where the water table depth is less than half the surface depression—that is, less than 1 foot (0.3 m) below land surface.

**Table 2:** Total Simulated Surface and Subsurface Fluxes (ft3/s) to Glacial System in Mukwonago Basin

Flux Above Water Table	Inflow	Outflow
Infiltration across land surface	44.58	
Rejected recharge^1^		1.15

Groundwater Flux within Basin	Inflow to Glacial Groundwater	Outflow from Glacial Groundwater	Net IN to Glacial Groundwater	Percent of Total Net IN
Recharge to water table	43.43		43.43	94.1
Lateral flow at basin boundary	10.37	7.63	2.74	5.9
		SUM=	46.17	100.0

	Inflow to Glacial Groundwater	Outflow from Glacial Groundwater	Net OUT from Glacial Groundwater	Percent of Total Net OUT
Bedrock/glacial exchange	0.91	3.82	2.91	6.3
Stream exchange	5.55	13.29	7.74	16.7
Lake discharge		17.70	17.70	38.2
Seepage to land surface in riparian and fen areas^1^		17.88	17.88	38.6
High capacity glacial well discharge		0.11	0.11	0.2
		SUM=	46.34	100.0

Percent error in‐out = 0.4%. Glacial system consists of layers 1, 2, and 3 of Mukwonago flow model.

1 Rejected recharge and seepage are routed to streams as groundwater runoff.

**Figure 7:**
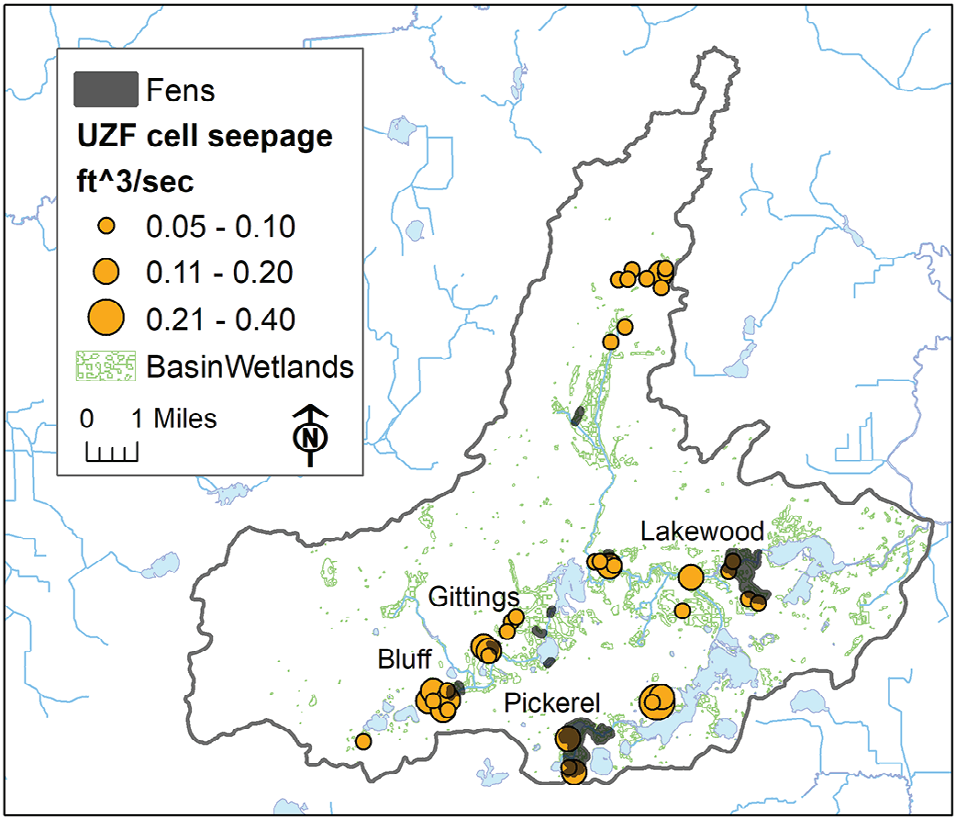
Simulated fen discharge in Mukwonago Basin.

The simulated fen seepage can be divided into the riparian portion occurring within SFR cells (63% of total seepage) and the portion occurring outside SFR cells (37% of total seepage). The nonriparian portion occurs over 114 basin cells. But only 40 of these cells—amounting to 230 acres (97 ha)—host seepage rates greater than the assumed “fen threshold” equal to 0.05 ft^3^/s (0.0014 m^3^/s). The fen seepage totals 4.1 ft^3^/s (0.117 m^3^/s). Most of the simulated seepage is associated with the upgradient sides of mapped fens in the basin (Figure 7). These are the fen areas that are most at risk if, for example, local pumping draws down the water table and reduces the availability of groundwater to the fens. Therefore, these may serve as “sentinel areas” for monitoring fen changes.

A sensitivity analysis was conducted on the relation of simulated seepage to assumed surface depression depth. The relation is modestly sensitive: reducing *D* from 2 feet (0.61) to 1 foot (0.30 m) reduced total seepage in fen cells (Figure 7) by 3.5%; increasing *D* to 3 feet (0.91 m) increased total fen seepage by 6.9%. With the lower surface depression depth, the fen distribution stayed the same as in Figure 7; for the higher surface depression depth, the fen area increased in area from 0.45% to 0.51% of the basin.

Whereas the seepage and fen solutions for the example application are not sensitive to the surface depression depth *D*, they are sensitive to the *K*_*v*_ assigned the surficial material at depths shallower than *D*. By default, this uppermost part of the flow system is assigned the same *K*_*v*_ values as the underlying material in model layer 1. However, the UZF package accepts a separate array to populate *K*_*v*_ values above the surface depression depth, an array which is used exclusively for the simulation of seepage and rejected recharge rates (and not used for calculation of saturated or unsaturated flow). To test the sensitivity of the solution to increased resistance to land surface discharge posed by this surficial part of the flow system, an array was inserted into UZF, with values everywhere one tenth the magnitude of those assigned the glacial material in layer 1. The effect of the change was to raise water‐table elevations in the upland parts of the basin by as much as 4 feet (1.2 m) and reduce seepage in the lowland areas from 18 ft^3^/s (0.51 m^3^/s) to 5 ft^3^/s (0.15 m^3^/s). Moreover, the area represented by fen cells (where seepage exceeds the assumed threshold of 0.05 ft^3^/s) dropped from 2.1% to 0.15% of the basin. The latter result is in much less agreement with the distribution of mapped fens than the former, indicating that the increased surficial resistance in the sensitivity run is probably unrealistic. In the present study this surficial K_v_ array was not tested explicitly through the PEST calibration process, but the sensitivity analysis suggests that it would be a good idea in future work to include it formally in the process as a way to improve the match to the observed distribution of fens.

To investigate the effect on fens of stresses from pumping, a series of simulations was performed where we inserted one of four hypothetical pumping wells, each pumping 100 gal/min (545 m^3^/d), and each located 1500 feet (457 m) from the nearest active seepage cell of a mapped fen (the location of the four wells, each corresponding to a separate simulation, are shown in Figure 5). The discharge at the wells was divided between model layers 1 (shallow glacial) and 2 (deep glacial) as a function of their respective saturated transmissivities. The overall loss of seepage due to steady‐state pumping at the mapped fens varies between 3 and 13% of their prepumping flows (Table 3A). For individual fen cells located 1500 feet (457 m) from the well, the losses range from 13% to 31% of prepumping flows, a relatively large fraction of the groundwater available to the fen (Table 3B). These losses correspond to 0.11 to 0.22 foot (3 to 6 cm) of water table drawdown. The range of seepage loss and drawdown is a function largely of local glacial sediment thickness and depth of pumping, local glacial sediment transmissivity, and the availability of other sources of water to cells from nearby streams and lakes.

**Table 3:** Pumping Scenario: Effect of Well Discharge on Seepage to Fens

A. Reduced Seepage at Named Fens Due to Glacial Pumping Well Discharging 0.223 ft^3^/s (=100 gal/min)				
Fen	Base Seepage (ft^3^/s)	Seepage with Pumping (ft^3^/s)	Well Discharge Derived from Fen %	Reduced Seepage to Fen %
Gittings	0.182	0.157	11%	−13%
Pickerel	1.760	1.670	41%	−5%
Bluff	1.169	1.133	16%	−3%
Lakewood	0.806	0.752	25%	−7%

B. Reduced Seepage to Active Fen Cell Closest to Well (1500 ft distant)				
Fen	Drawdown at Closest Fen Cell, Foot	Base Seepage (ft^3^/s)	Seepage with Pumping (ft^3^/s)	Reduced Seepage at Closest Fen Cell %
Gittings	0.18	0.155	0.135	−13%
Pickerel	0.19	0.174	0.144	−17%
Bluff	0.11	0.079	0.068	−14%
Lakewood	0.22	0.051	0.035	−31%

For location of fens and pumping wells, see Figure 5.

## Limitations

A key limitation of the method is that the steady state assumption does not incorporate the seasonal importance of water level elevation in the fen root zone. Steady‐state assumptions are consistent with stability of groundwater discharge to fens, but the neglect of evapotranspiration and other near‐surface and root‐zone factors implies that the distribution of fens output by the model is only an approximation of a very dynamic process (Hunt et al. 2008). The computational burden of substituting a transient for steady‐state flow model simulation is high, and requires that the UZF package contain unsaturated flow parameters to explicitly simulate unsaturated flow.

Steady‐state infiltration is best input to the model as the annual precipitation net of evapotranspiration and storm runoff (estimated, for example by a soil‐water balance method). In this sense, infiltration corresponds to the water percolating below the root zone, but the method requires that the top of the cell correspond to the land surface rather than the bottom of the root zone.

For the application presented in this paper, an important limitation is the size of the lateral grid spacing: 500 feet (152 m). The resulting cell area is equal to 5.7 acres (2.3 ha). Whereas this spacing is fine enough to represent the entire surface water network including first‐order streams (Feinstein et al. 2018), it is too coarse to always differentiate mapped fen locations, which in the Mukwonago area can be as small as 2 acres (0.8 ha). It also means that the land surface elevation at a cell, and resulting surface depression depth, crucial to the method, is an average value that may obscure subtle but important topographic variations. The present application also took no account of observed peat formations and their possible effect on the local *K*_*v*_ term associated with seepage discharge from the shallow groundwater. A finer scheme of layering would be necessary to properly consider the peat thickness even if it could be mapped and its permeability evaluated.

A limitation particular to the application of the method presented here is the choice of a “fen threshold.” This threshold seepage (which can be converted into a rate by dividing by the model cell area) should ideally be tied to an estimate of the rate of groundwater flow needed to support the fens under study during their productive seasons. This rate is expected to be a function of fen vegetation and type, requiring a more ecohydrological characterization.

Finally, the reliability of the method depends on the accuracy of inputs such as the land surface elevation, the average depth of surface undulations, the geometry of the surface water network, and the spatial distribution of infiltration below the root zone. Among these inputs the infiltration rate is the most difficult to estimate and generally requires application of a special technique—for example, the Soil Water Balance model (Westenbroek et al. 2009).

## Discussion

The use of the UZF package to simulate surface leakage allows for more realistic flow simulations because it can account for discharge mechanisms that are neglected when groundwater is forced to circulate to a stream or lake represented by a head‐dependent boundary condition rather than to discharge to the land surface. When land‐surface seepage is excluded as a discharge mechanism, the flow system tends to become over‐pressurized in lowland areas, the extra pressure providing the gradient needed to carry the groundwater beyond it natural discharge location to a boundary surface water feature. This form of structural error can lead to either spurious groundwater flooding in lowland areas or to miscalibration of model parameters to avoid simulating groundwater flooding.

The use of UZF to simulate surface leakage is a readily applied method which overcomes the problem of over‐pressurization. The approach presented here is deemed simple because it neglects the range of unsaturated flow options that are implemented in a standard UZF application and because of the ease with which it is applied to an existing steady‐state MODFLOW model. As noted above, it has many limitations. Some flow terms are neglected—not only unsaturated and transient flow, but also possible lateral groundwater and transient runoff contributions to wetland discharge. *If* despite these limitations, the method allows mapped fens to be reproduced as a function of a few major model inputs (some of which are easily obtained, notably the cell‐by‐cell topography, and some of which must be calculated or calibrated such as the vertical hydraulic conductivity of the surficial material), then there is a good chance that a fairly robust representation of the fen distribution can be achieved. *If* during calibration the simulated fen locations compare favorably to mapped fens, then it is reasonable to use the model as a screening tool for identifying where fens might be most vulnerable to development by pumping or land use changes or climate change. This approach has been adopted by The Nature Conservancy (TNC) in the Mukwonago Basin where a decision support system is under design by the TNC to rapidly evaluate (by means of the MODFLOW model documented in the online Supporting Information) the effects of different levels of pumping on both the health of basin fens and the availability of groundwater discharge to basin streams and lakes (Miller et al. 2014).

**Figure 8:**
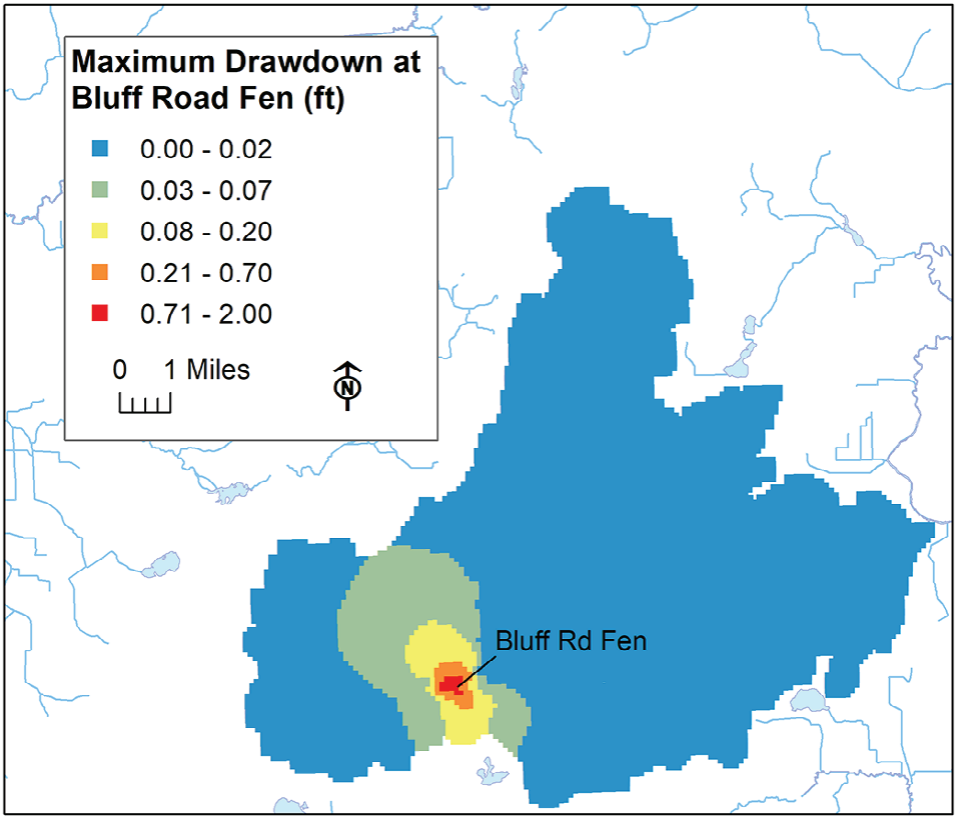
Maximum drawdown potential at Bluff Road Fen from single glacial well pumping 100 gal/min located anywhere within Mukwonago Basin.

The decision support evaluation process involves placing a single well in a model row/column location within the studied basin and having it pump at a selected rate from the shallow glacial layers (the withdrawal from each layer set proportional to the layer's saturated transmissivity). Individual simulations are conducted for each basin cell location (the Mukwonago Basin encompasses 8859 row/column locations). The combined results allow the effect of pumping on a specific resource location (such as a named fen) to be mapped in terms of the flux reduction or drawdown attributable to a shallow well anywhere in the basin. For example, the vulnerability of the Bluff Road fen to pumping can be evaluated in this way. The drawdown results for the fen predict that a glacial well pumping 100 gal/min (545 m^3^/d) within a distance of about 1500 feet (500 m) from the fen will provoke as much as a 0.7 feet (20 cm) drop in head (Figure 8), a reduction in water‐table elevation that could pose a risk to the health of the fen, as suggested in Aldous and Bach 2014.

## Conclusions

1. Simply adding an implementation of UZF (without unsaturated flow parameters) to a steady‐state regional model can provide a representative simulation of areas with groundwater seepage to fens. It is not necessary to “rig” the model by specifying explicit hydrologic boundary conditions or fen location/properties/processes.
2. As an alternative to blanketing the model domain with land‐surface drains, the UZF method offers computational advantages tied to the consideration of surface depressions and surficial hydraulic conductivity, and record‐keeping advantages tied to output arrays of subgridded flows and groundwater runoff.
3. Model results are driven by land surface elevations in low‐lying areas—and topography is typically a relatively high accuracy model input. For screening applications, simulations to address the distribution of fens and their vulnerability to pumping do not necessarily need the complexity of transient flow, unsaturated flow, peat layers, gravel beds, and vertical structures giving rise to preferential flow paths.
4. Fens and high‐capacity pumping depend on the larger‐scale groundwater system—thus, the model should nest local in regional flow systems. For managerial purposes, it may be better to simplify or neglect intra‐wetland processes (like transient evapotranspiration or lateral overland flows) and retain good simulation of regional and local flows to make forecasts about the response of fens to development. This response can be evaluated with an appropriately constructed groundwater flow model in terms both of drawdown at the water table and reduction of seepage to the land surface.

## Authors' Note

The author(s) does not have any conflicts of interest or financial disclosures to report.

## Supporting information


**Figure S1.** Mukwonago Basin study area.
**Figure S2.** Model domain and boundary conditions.
**Figure S3.** Hydrostratigraphic section through Mukwonago Basin.
**Figure S4.** Model boundary conditions in vicinity of Mukwonago Basin.
**Figure S5.** Logs fully penetrating glacial thickness, showing interpolated glacial texture class, in vicinity of Mukwonago Basin.
**Figure S6.** Model saturated glacial thickness in vicinity of Mukwonago Basin.
**Figure S7.** Model composite glacial horizontal hydraulic conductivity in vicinity of Mukwonago Basin.
**Figure S8.** Model saturated bedrock thickness in vicinity of Mukwonago Basin.
**Figure S9.** Model composite bedrock horizontal hydraulic conductivity in vicinity of Mukwonago Basin.
**Figure S10.** Model Infiltration rates in vicinity of Mukwonago Basin.
**Figure S11.** High‐capacity glacial and bedrock pumping wells in Mukwonago Basin.
**Figure S12.** Head and baseflow calibration targets in vicinity of Mukwonago Basin.
**Figure S13.** Calibration scatter plots for stream baseflow and groundwater heads.
**Figure S14.** Simulated water table in vicinity of Mukwonago Basin.
**Figure S15.** Simulated water budget for Mukwonago Basin—all layers.
