# Supplementary material for: A Simple Method for Simulating Groundwater Interactions with Fens to Forecast Development Effects
Source: Ground Water. 2019 Aug 20;58(4):524–34. doi: 10.1111/gwat.12931 (PMC7383665; doi:10.1111/gwat.12931)
Supplement: Supplementary file 1 — Figure S1. Mukwonago Basin study area. Figure S2. Model domain and boundary conditions. Figure S3. Hydrostratigraphic section through Mukwonago Basin. Figure S4. Model boundary conditions in vicinity of Mukwonago Basin. Figure S5. Logs fully penetrating glacial thickness, showing interpolated glacial texture class, in vicinity of Mukwonago Basin. Figure S6. Model saturated glacial thickness in vicinity of Mukwonago Basin. Figure S7. Model composite glacial horizontal hydraulic conductivity in vicinity of Mukwonago Basin. Figure S8. Model saturated bedrock thickness in vicinity of Mukwonago Basin. Figure S9. Model composite bedrock horizontal hydraulic conductivity in vicinity of Mukwonago Basin. Figure S10. Model Infiltration rates in vicinity of Mukwonago Basin. Figure S11. High‐capacity glacial and bedrock pumping wells in Mukwonago Basin. Figure S12. Head and baseflow calibration targets in vicinity of Mukwonago Basin. Figure S13. Calibration scatter plots for stream baseflow and groundwater heads. Figure S14. Simulated water table in vicinity of Mukwonago Basin. Figure S15. Simulated water budget for Mukwonago Basin—all layers. [file GWAT-58-524-s001.docx]

**Supporting Information**

For Journal of Ground Water article titled *“A Simple Method for Simulating Groundwater Interactions with Fens to Forecast Development Effects”*

**Authors:**

Daniel T. Feinstein, U.S. Geological Survey, [dtfeinst@usgs.gov](mailto:dtfeinst@usgs.gov)

David J. Hart*, Corresponding Author:* Wisconsin Geological and Natural History Survey, University of Wisconsin-Madison, 3817 Mineral Point Road, Madison, WI 53705-5100; 608 262-2307; fax: 608 262-8086; [david.hart@wisc.edu](mailto:david.hart@wisc.edu)

Sarah Gatzke, The Nature Conservancy, Madison, WI; [sarah.gatzke@tnc.org](mailto:sarah.gatzke@tnc.org)

Randall J. Hunt, U.S. Geological Survey, Middleton, WI; [rjhunt@usgs.gov](mailto:rjhunt@usgs.gov)

Richard G. Niswonger, U.S. Geological Survey, Menlo Park, CA; [rniswon@usgs.gov](mailto:rniswon@usgs.gov)

Michael N. Fienen, U.S. Geological Survey, Middleton, WI; [mnfienen@usgs.gov](mailto:mnfienen@usgs.gov)

**Abstract:**

Protection of fens - wetlands dependent on groundwater discharge - requires characterization of groundwater sources and stresses. Because instrumentation and numerical modeling of fens is labor intensive, easy-to-apply methods that model fen distribution and their vulnerability to development are desirable. Here we demonstrate that fen areas can be simulated using existing steady-state MODFLOW models when the Unsaturated Zone Flow (UZF) package is included. In cells where the water table is near land surface, the UZF package calculates a head difference and scaled conductance at these “seepage drain” cells to generate average rates of vertical seepage to the land. This formulation, which represents an alternative to blanketing the MODFLOW domain with drains, requires very little input from the user because unsaturated flow-routing is inactive and results are primarily driven by easily-obtained topographic information. Like the drain approach it has the advantage that the distribution of seepage areas is not pre-determined by the modeler, but rather emerges from simulated heads. Beyond the drain approach, it takes account of intra-cell land surface variation to explicitly quantify multiple surficial flows corresponding to infiltration, rejected recharge, recharge and land-surface seepage. Application of the method to a basin in southeastern Wisconsin demonstrates how it can be used as a decision-support tool to first, reproduce fen distribution and, second, forecast drawdown and reduced seepage at fens in response to shallow pumping.

***

The Supporting Information presented here accompanies the article: *“A Simple Method for Simulating Groundwater Interactions with Fens to Forecast Development Effects”* (Feinstein, Hart, Gatzke, Hunt, Niswonger, and Fienen, 2019; also, USGS data release: Jones and Feinstein, 2019). It documents the Mukwonago groundwater flow model in terms of the following elements:

- role of MODFLOW simulation in the overall study p. 3
- brief account of the hydrologic system under investigation p. 6
- MODFLOW version p. 8
- grid discretization and layering p. 8
- model boundary conditions p. 9
- hydrostratigraphic properties of groundwater system p. 11
- aquifer stresses p. 17
- simulation of seepage to land surface p. 20
- calibration process and summary of output p. 21
- model limitations p. 26
- acknowledgments p. 27
- references p. 27

The distinguishing aspects of the modeling approach targeting fens in the Mukwonago basin are highlighted in the text of the published article. This Supporting Information is intended to document the development of the model in a more thorough way.

The text is accompanied by the following figures:

Figure S1. Mukwonago Basin study area.

Figure S2. Model domain and boundary conditions.

Figure S3. Hydrostratigraphic section through Mukwonago Basin.

Figure S4. Model boundary conditions in vicinity of Mukwonago Basin.

Figure S5. Logs fully penetrating glacial thickness, showing interpolated glacial texture class,

in vicinity of Mukwonago Basin.

Figure S6. Model saturated glacial thickness in vicinity of Mukwonago Basin.

Figure S7. Model composite glacial horizontal hydraulic conductivity in vicinity

of Mukwonago Basin.

Figure S8. Model saturated bedrock thickness in vicinity of Mukwonago Basin.

Figure S9. Model composite bedrock horizontal hydraulic conductivity in vicinity of

Mukwonago Basin.

Figure S10. Model Infiltration rates in vicinity of Mukwonago Basin.

Figure S11. High-capacity glacial and bedrock pumping wells in Mukwonago Basin.

Figure S12. Head and baseflow calibration targets in vicinity of Mukwonago Basin.

Figure S13. Calibration scatter plots for stream baseflow and groundwater heads.

Figure S14. Simulated water table in vicinity of Mukwonago Basin.

Figure S15. Simulated water budget for Mukwonago Basin – all layers.

1. Role of MODFLOW simulation in the overall study.

The steady-state Mukwonago groundwater flow model (MGWF model), archived in the USGS data release Jones and Feinstein, 2019, is derived from a parent steady-state model of the Upper Fox basin (UPFOX model), documented in Feinstein et al., 2018 and Haserodt et al., 2018. The UPFOX model, in turn, is an inset model extracted from a grandparent transient model of the Lake Michigan basin (LMB model), which is documented in Feinstein et al., 2010. The UPFOX extraction corresponds to 2005 conditions in the LMB model.

The MGWF model is designed to simulate the shallow and deep circulation of groundwater in the Mukwonago Basin (fig. S1), and the interaction of shallow groundwater with surface water, notably with groundwater-fed wetlands. The MGFW model, covering parts of three counties in southeastern Wisconsin, is updated with respect to the parent UPFOX model with respect to 1) the representation of the surface-water system within the Mukwonago Basin and surrounding areas, 2) the hydraulic conductivity of the shallow glacial deposit. These changes are designed to strengthen the model’s ability to simulate groundwater-surface water interactions.

Importantly, the functionality of the MGWF model is expanded to include groundwater discharge to the land surface as seepage, allowing the model to simulate groundwater-fed wetlands (fens) as an output rather than constraining their locations through insertion of a fixed boundary condition. See the published article for more discussion of this feature. Including groundwater discharge to the land surface is crucial to implementing the key objective of the modeling effort: to evaluate fen discharge and the vulnerability of fens to shallow pumping.


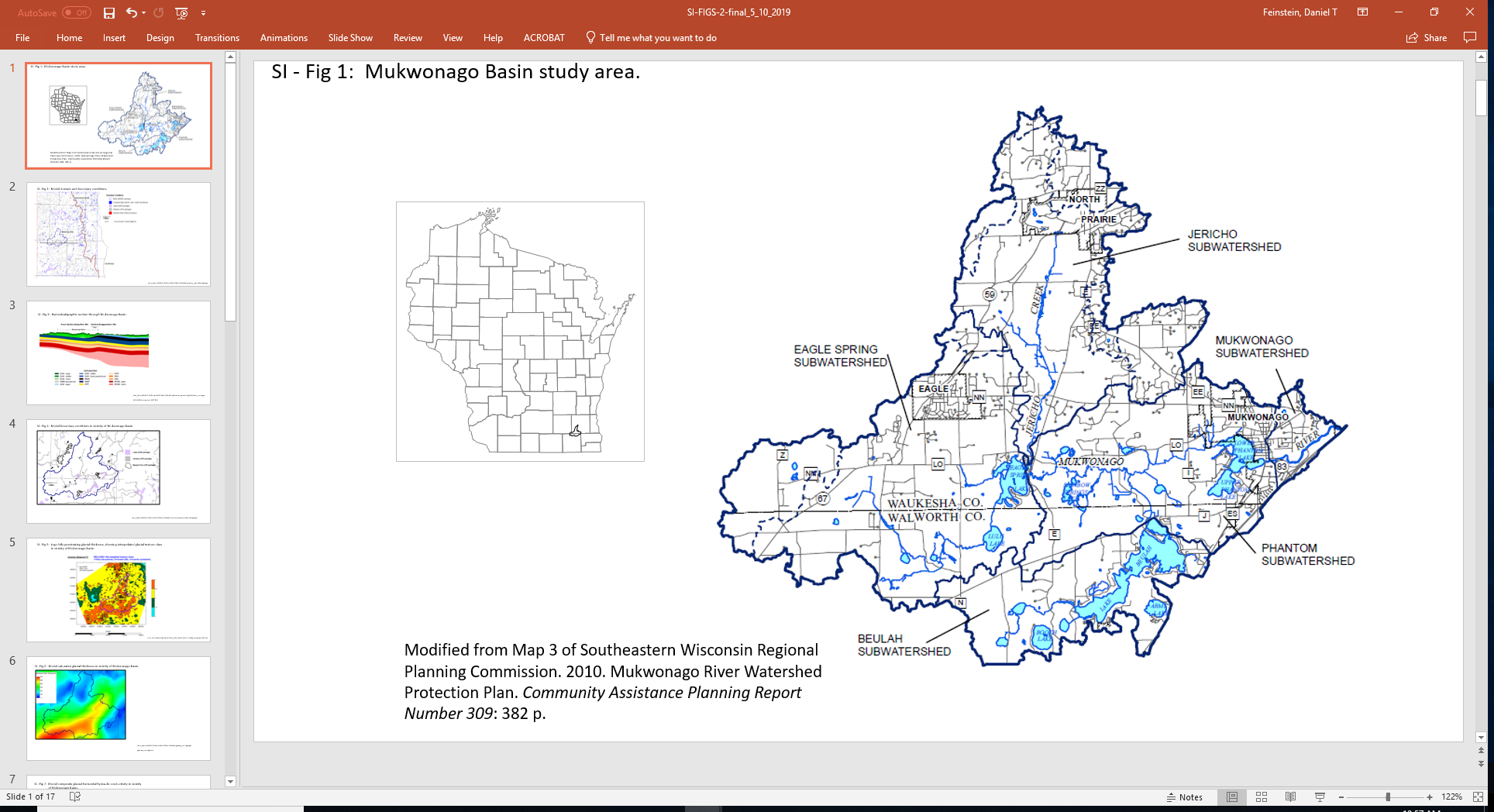


**Figure S1. Mukwonago Basin study area.**


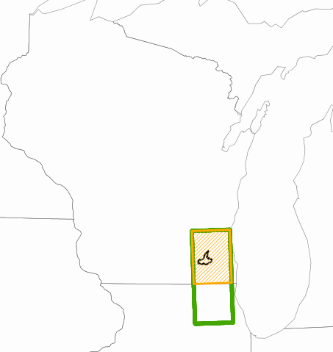

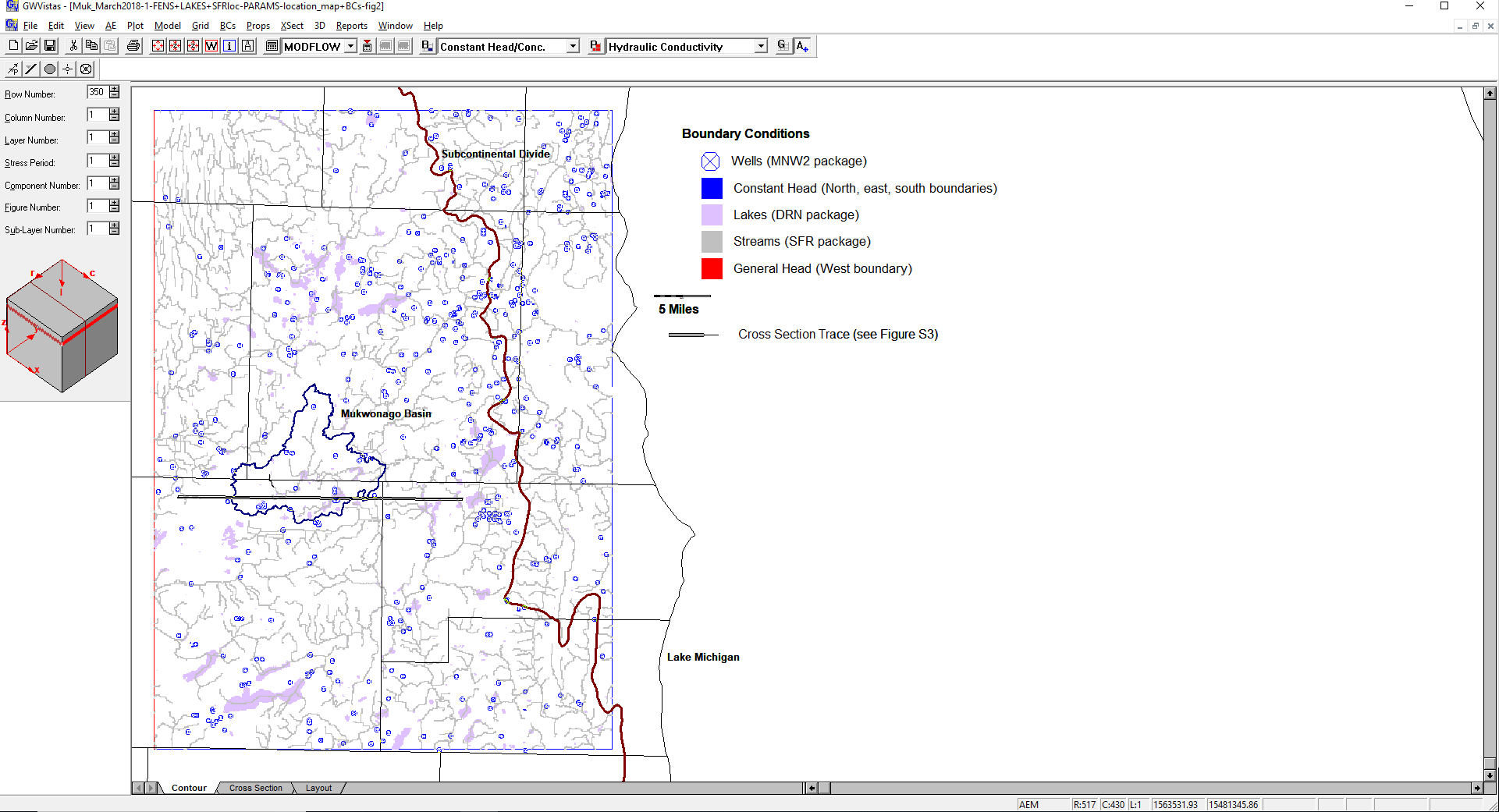


Thumbnail map

Illinois

Wisconsin

**Wisconsin/Illinois state line**

**Figure S2. Model domain and boundary conditions.** Note: The thumbnail map shows the relation of the MGWF model domain (stippled area) in the northern half of the parent UPFOX model domain (entire rectangle). The Mukwonago Basin is outlined in both figures.

2. Brief account of the hydrologic system under investigation.

The Mukwonago Basin covers an area of 86.21 mi^2^ (223.3 km^2^) in southeastern Wisconsin (Fig. S1), overlapping parts of Waukesha, Walworth, and Jefferson counties. Its drainage area corresponds to Hydrologic Unit Code 0712000602 (HUC10 scale), nested within the Fox River Basin, which in turn is tributary to the Mississippi River (<https://dnr.wi.gov/water/watershedsearch.aspx>). The Mukwonago Basin constitutes the nearfield of the MGWF model. Its groundwater is in communication with groundwater in surrounding topographic basins which constitute the farfield of the model (Fig. S2).

The hydrogeologic setting of the MGWF model consists of unconsolidated glacial deposits (with some alluvial material) over sedimentary bedrock. The following account of the hydrogeology of southeastern Wisconsin is modified from Appendix 1.2.2 of Feinstein et al., 2010:

The bedrock hydrostratigraphy of southeastern Wisconsin consists of Paleozoic

sedimentary units generally thickening to the east. In most places, Pleistocene deposits of till, sand and gravel, or lake sediment cover the bedrock units, and bedrock outcrops are rare. Cambrian-Ordovician units rest on the Precambrian crystalline basement rocks, which transmit little water and form the bottom boundary to the aquifer system. In ascending order, the major water-producing units of the deep aquifer are sandstones of the Mount Simon Formation (MTSM), the Ironton-Galesville Formation (IRGA), and the St. Peter Formation (STPT).

Between the Mount Simon Formation and the Ironton-Galesville Formation lies the Eau Claire Formation (EACL), composed of shale and sandstone. A laterally extensive shaly zone within the Eau Claire Formation forms a confining unit over much of southern Wisconsin. Rocks between the Ironton-Galesville and St. Peter Formations are identified with the Prairie du Chien and Franconia Formation (PCFR). They can form a leaky confining unit and are made up of interbedded sandstone, shale, siltstone and dolomite. Overlying the St. Peter Formation, dolomite of the Sinnipee Group (SNNP and shale of the Maquoketa Formation (MAQU) together make up a major confining unit between deep and shallow aquifers. The hydraulic properties of the Sinnipee Group dolomite depend on whether it is overlain by the Maquoketa. Where it is not, and forms the uppermost bedrock unit, it is highly weathered and relatively permeable. Deep wells are generally cased through the Maquoketa Formation and open from the Sinnipee Group to the St. Peter Formation or lower in the deep part of the flow system. The Silurian-Devonian aquifer (SLDV), predominately dolomite, and the unlithified Pleistocene materials (GLAC), made up of till, sand and gravel, and lake sediment from several glacial lobes, constitute important shallow sources of public and domestic water supply.

All southeastern Wisconsin sedimentary rocks dip gently to the east and south, and erosion at the bedrock surface has truncated the uppermost units so that the Maquoketa Formation and overlying rocks are present in only the eastern part of southeastern Wisconsin. Unlithified Quaternary deposits blanket these rocks at thicknesses of less than 25 ft (7.6 m) to more than 400 ft (122 m). They are thicker in areas where the bedrock surface is incised. Ancient drainage cut down through the shallowest bedrock penetrating the Silurian-Devonian dolomite, the shale of the Maquoketa Formation and the dolomite of the Sinnipee Group, resulting in buried valleys filled with Quaternary deposits. Where dolomite of the Sinnipee Group is the uppermost bedrock unit in the west, it forms a minor part of the deep aquifer system. Quaternary deposits can form aquifers in areas where they are sufficiently thick and are dominated by sand and gravel, but they act as confining units near Lake Michigan, where they are primarily clays and silts.

The glacial deposits in the vicinity of the Mukwonago Basin (Fig. S3) vary between zero and 300 ft (91 m). The underlying bedrock sequence of sedimentary rocks varies between about 1000 ft (305 m) and 2000 ft (610 m). The bedrock is capped by the Silurian-Devonian aquifer and Maquoketa Shale confining unit in the eastern part of the Basin, whereas the uppermost bedrock unit is the weathered Sinnipee dolomite in its western part.


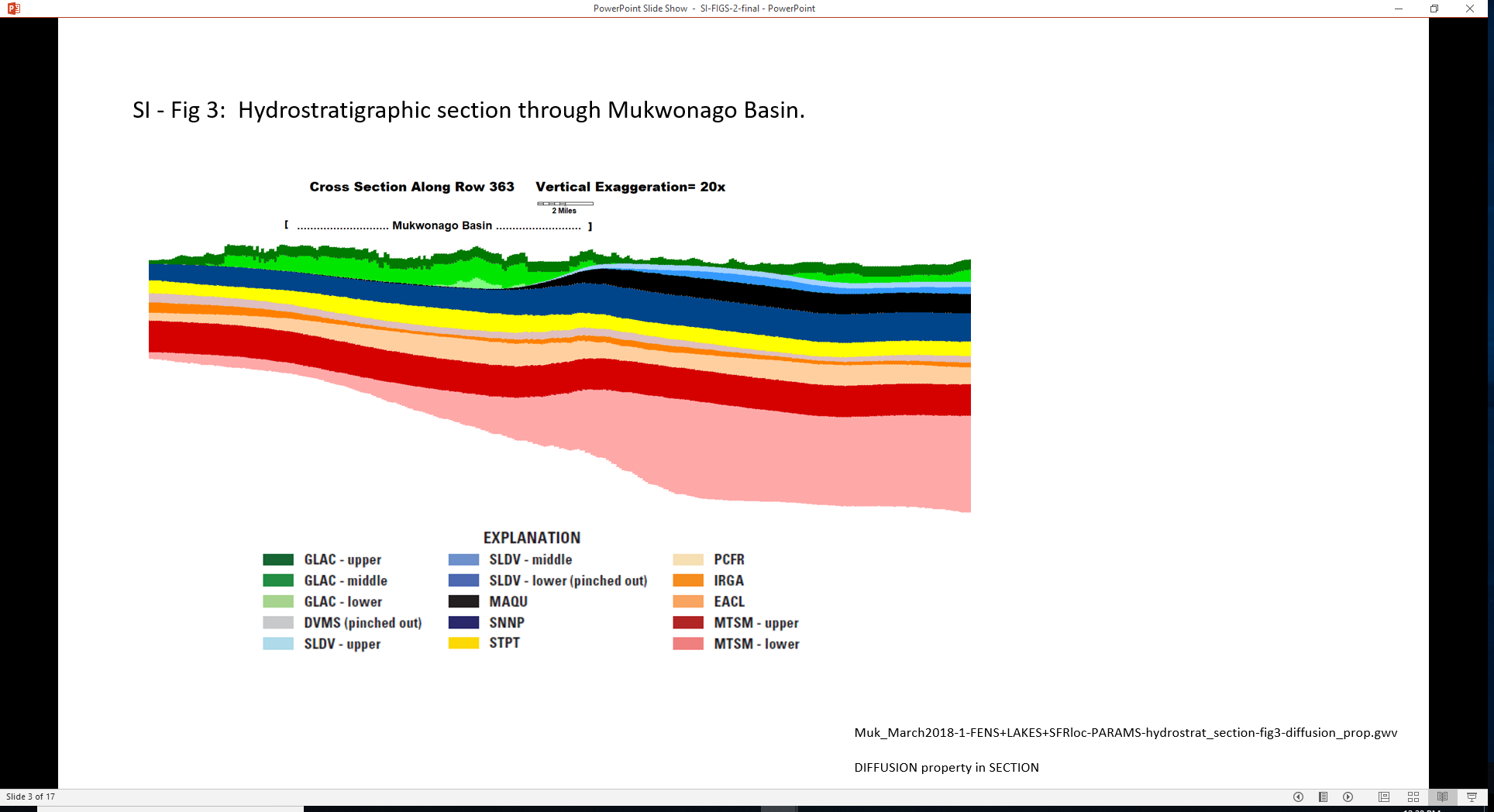


**Figure S3. Hydrostratigraphic section. See cross section trace in Figure S2.**

3. MODFLOW version

The MGWF model was run with the MODFLOW-NWT code (Niswonger and others, 2011). MODFLOW-NWT incorporates a Newton-Raphson code in place of the Picard method that was previously standard to MODFLOW. MODFLOW-NWT overcomes difficulties involving drying and rewetting nonlinearities of the unconfined groundwater-flow equation and yields robust values for water-table elevations that may not converge with the Picard method (Feinstein et al., 2012). The focus of the MGWF model is on the shallow, unconfined part of the groundwater flow system, particularly the interaction of surface water and wells with the glacial aquifer system. The MODFLOW-NWT code is well suited for reliably addressing this type of problem.

Version 1.1.4 of MODFLOW-NWT (dated 4/1/2018) was used for the MGWF model. It is based on MODFLOW-2005 version 1.12.0 (dated 2/3/2017).

4. Grid discretization and layering.

The main motive for extracting the MGWF model from the UPFOX parent model was to reduce the solution space for the child model. The UPFOX model spans parts of southeastern Wisconsin and northeastern Illinois (Feinstein et al., 2018). The MGWF model is limited to the northern half of the UPFOX domain bounded at the south by the Wisconsin/Illinois state line (Fig. S2).

The MGWF model was extracted from the parent UPFOX model without further refinement to either the lateral or vertical grid spacing. The grid spacing remains 500 ft (152.4 m) on a side and the thickness of hydrostratigraphic units (Fig. S3) is inherited from the parent model.

The MGWF model consists of 430 columns, 600 rows, and 15 layers. Layers 4-15 correspond to bedrock units. The top three layers represent the glacial deposits. The layering for these shallow layers follows the following logic (Feinstein et al., 2018):

- Layer 1 extends from land surface to a maximum depth of 100 ft; if the bottom of the glacial deposits is at a depth less than 100 ft below land surface, then the bottom of layer 1 is at that depth.
- If the glacial thickness is greater than 100 ft, then layer 2 extends from the 100-ft depth to a maximum depth of 300 ft; otherwise layer 2 is pinched.
- If the glacial thickness is greater than 300 ft, then layer 3 extends from the 300-ft depth to top of bedrock; otherwise layer 3 is pinched.

Pinched glacial cells are assigned a thickness of 0.1 ft and given the properties of the overlying layers. Bedrock cells can also be pinched (for example, Silurian layers in the western part of the Mukwonago Basin). Pinched bedrock cells are assigned a thickness of 0.2 ft.

The model land-surface elevation values at the 500-ft grid spacing for the MGWF are derived from the National Elevation Dataset (U.S. Geological Survey, 2014). The mean elevation at 10-meter grid spacing are computed for the area of each inland row/column location and assigned to the top of layer 1.

5. Model boundary conditions.

The MGWF model is extracted from part of the parent UPFOX model, but maintains the grid spacing and many of the boundary conditions from the parent model. The edge constant heads cells assigned to all layers along the northern and eastern extent of the MGWF model domain are identical to the boundary condition input for the UPFOX inset model, which was, in turn, extracted from the LMB regional model. The constant head condition to the south at the Wisconsin/Illinois state line corresponds to the steady-state solution of the UPFOX model for the model row coincident with the state line. The northern, eastern, and southern edge boundary conditions are distant from the Mukwonago Basin (Fig. S2), separated by several major topographic and groundwater divides. It is reasonable to assume that fixed head boundary condition at these locations (for both the shallow and deep parts of the groundwater flow system) does not bias the nearfield groundwater flow solution.

The western edge of the MGWF domain is relatively close to the Mukwonago Basin (Fig. S2). The western side of the Basin is about 7 miles (11.3 km) east of the edge of the domain. To avoid compromising the model solution by imposing fixed heads along this edge, the constant head cells in the parent UPFOX model were replaced by cells hosting a General Head Boundary (GHB) condition. The GHB condition effectively moves the fixed head some distance beyond the model edge and inserts a conductance term between the distant location and the model edge to account for the intervening head loss. The difference between the fixed external head and the model solution at the edge boundary is multiplied by the conductance term to yield a flux into or out of the model domain. In this way, the magnitude of the flux boundary condition adjusts to the stresses inside the model domain rather than imposing a fixed condition.

In the case of the MGWF model, the external head location was set 15,000 ft (4572 m) west of the western edge of the model. The fixed heads for the GHB cells were extracted from a column of the LMB grandparent model, whose grid extends farther west than either the UPFOX or MGWF models, but which shares the same layering scheme as its descendants. The LMB heads are extracted from the model solution for the year 2005. The conductance term for each model layer is equal to the harmonic mean of the horizontal hydraulic conductivity assigned to LMB cells in the stretch between the external north/south GHB condition and the western edge of the MGWF model).

Streams within the MGWF model, including the Mukwonago Basin, were simulated with the Streamflow Routing (SFR2) package developed by Niswonger and Prudic (2005). The SFR2 package routes water from upstream reaches to downstream reaches to accumulate flow and solve for water level in the stream. Only groundwater discharge to the streams (base flow) is considered in the model simulations. The contribution of overland flow is excluded. In this sense, the result of the simulation corresponds to low-flow conditions in streams, generally encountered during the season between August and October.

The formulation of the SFR2 input is the same as that described in Feinstein et al. (2018) for the parent UPFOX model. The routing of the stream network was redone for the MGWF model to accommodate its smaller inset area. The stream network is shown in Figure S4.

As in the LMB and UPFOX models, surface-water bodies such as lakes are represented by the Drain (DRN) package. DRN cells only receive groundwater discharge, they do not contribute water to the aquifer system. It is convenient to use the DRN package to represent surface-water bodies to avoid perched lakes and wetlands acting as spurious sources of water. More details on the formulation of the DRN package, including the method for setting the lake stages, are given in Feinstein et al. (2018.). Whereas the parent models only apply drains to represent water bodies greater than 20 acres (8.1 hectares), the MGWF model hosts water bodies as small as small as two acres in size. The DRN cells representing lakes are shown in Figure S4. Most, but not all, of these features are “discharge lakes, that is, they are in connection with the stream network.


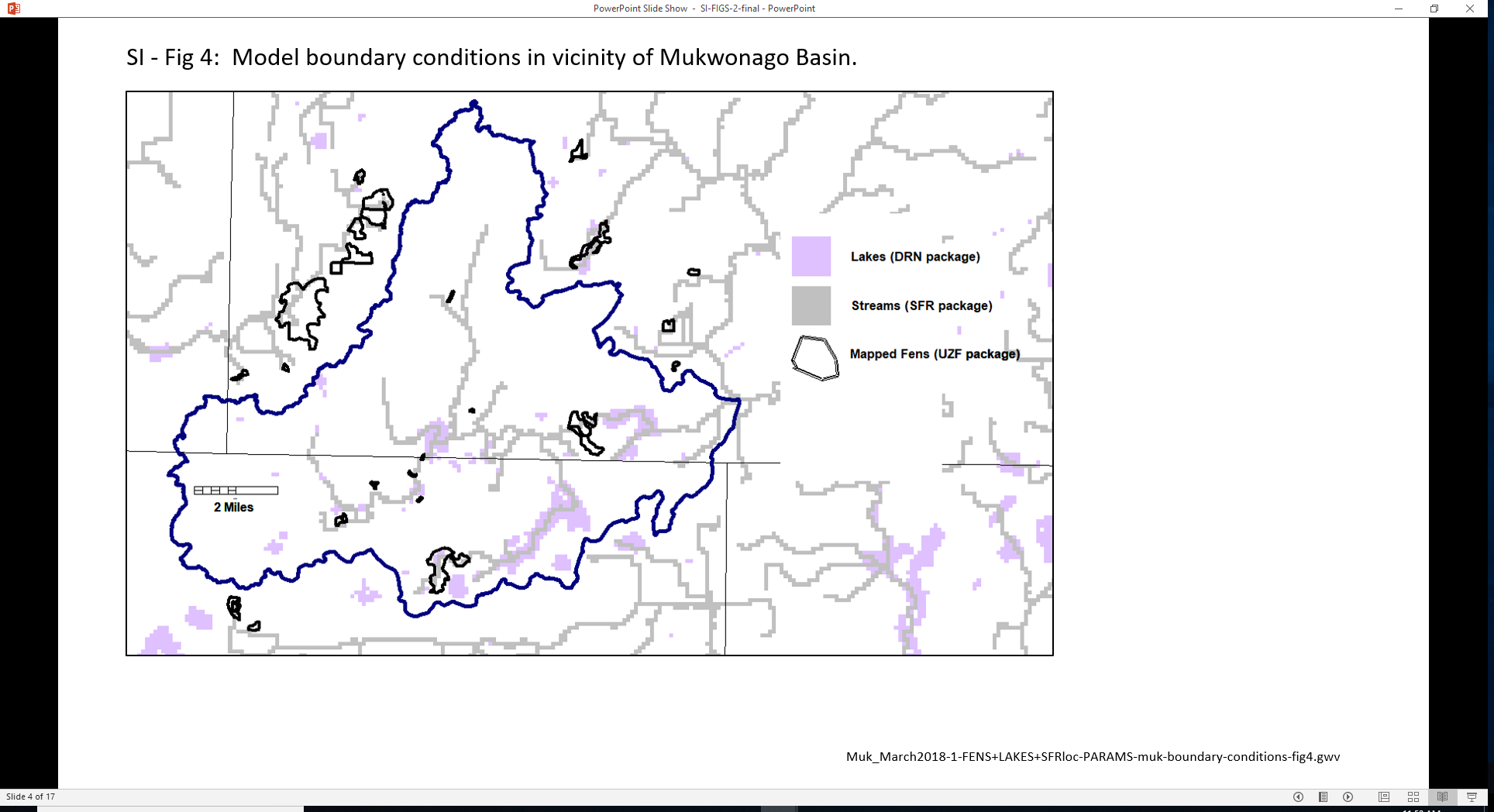


**Figure S4. Model boundary conditions in vicinity of Mukwonago Basin.** Note: the blue boundary outlines the Mukwonago Basin.

6. Hydrostratigraphic properties of groundwater system.

The horizontal hydraulic conductivity (K_h_) and vertical hydraulic conductivity (K_v_) assigned to the bedrock units (layer 4 to layer 15) of the MGWF model are inherited from the parent UPFOX model. The bedrock hydraulic conductivity values are organized by zones. The glacial K_h_ and K_v_ values for the farfield of the MGWF model, varying on a cell by cell basis, are also inherited from the UPFOX model. The data underlying the bedrock and glacial hydraulic conductivity assignments and the calibration process used to adjust them are described in Feinstein et al., 2010 and, also in Feinstein et al., 2018.

The MGWF model was updated with respect to the parent UPFOX model by smoothing the shallow glacial K_h_ and K_v_. The UPFOX K_h_ and K_v_ values varied on 5000 ft grids inherited from the grandparent LMB model, creating a blocky texture to the MGWF model. The coarse grid created step changes in the K_h_ and K_v_ values that could create unrealistic discharge or refraction of flow at the step changes. A 16,400-ft focal point average was applied to smooth the step changes. The length scale of the focal average was chosen to remove the step changes and preserve the approximate global values, length scale and extent of the texture classes in the texture mapping of the glacial sediments

(Fig. S5). Given the lack of data for parameter refinement, the blocky assignment of K_h_ and K_v_ values to the bedrock layers was left unchanged from the values assigned the parent UPFOX model.

The thickness and hydraulic conductivity assigned to the aquifers and confining beds which make up the shallow and deep parts of the groundwater flow system combined with the model boundary conditions (see Section 5 above) and stresses (see Section 7 below) to determine the steady-state solution of the MGWF model. The total glacial thickness and the thickness-weighted (that is, composite) K_h_ of the glacial layers are shown in Figures S6 and S7 for the model area in vicinity of the Mukwonago Basin. Similarly, the total bedrock thickness and the composite K_h_ of the bedrock layers are shown in Figures S8 and S9. In all cases the thicknesses correspond to the **saturated** thickness of the glacial and bedrock deposits, defined as the thicknesses below the simulated water table produced by the calibrated model solution (see Section 9, below). Note that glacial deposits are missing over several small areas within the Mukwonago Basin, in which case the water table elevation falls within the uppermost bedrock unit present (occurring over 3.5% of the Mukwonago Basin area).

Average (calibrated) values within the Mukwonago Basin are as follows:

Saturated glacial thickness = 155 ft = 47.2 m

Saturated glacial Kh = 24.58 ft/day = 7.49 m/day

Saturated glacial transmissivity = 3949 ft^2^/day = 366.9 m^2^/day

Saturated bedrock thickness = 1345 ft = 410.0 m

Saturated bedrock Kh = 1.17 ft/day 0.357 m/day

Saturated bedrock transmissivity = 1584 ft^2^/day 147.2 m^2^/day


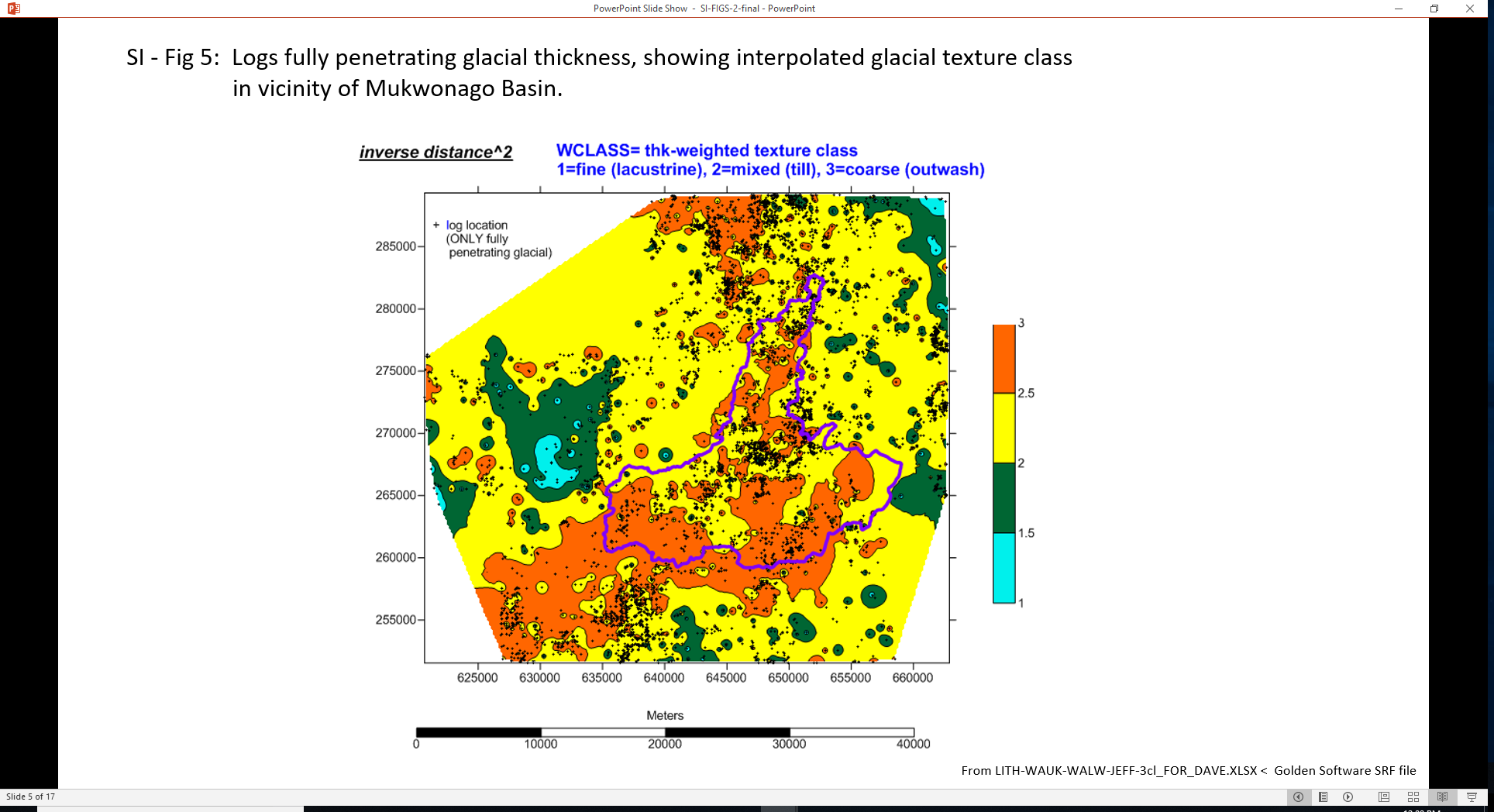


**Figure S5. Logs fully penetrating glacial thickness, showing interpolated glacial texture**

**class, in vicinity of Mukwonago Basin.** Note: the purple boundary outlines the

Mukwonago Basin.


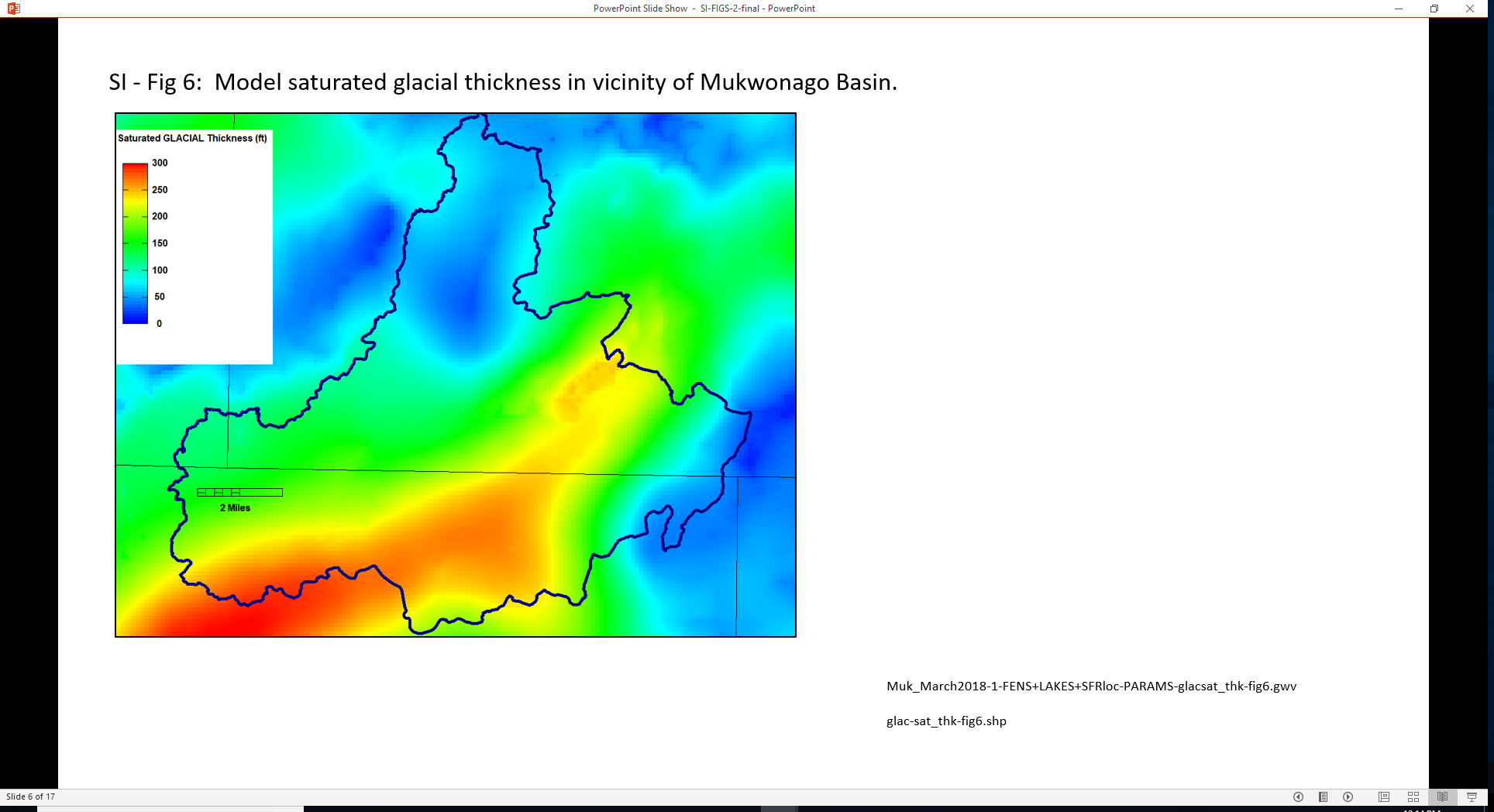


**Figure S6. Model saturated glacial thickness in vicinity of Mukwonago Basin**. Note: the blue boundary outlines the Mukwonago Basin.


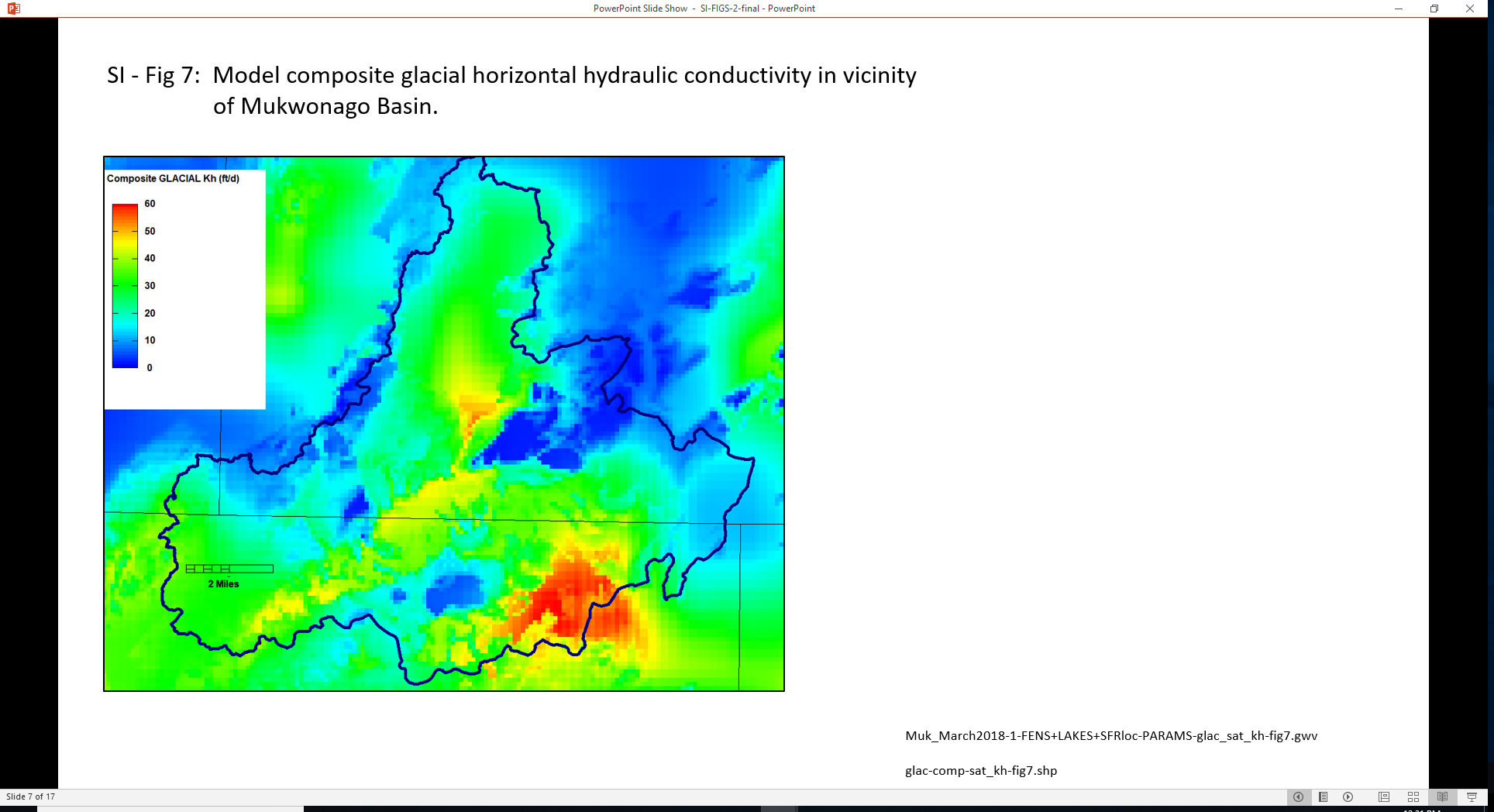


**Figure S7. Model composite glacial horizontal hydraulic conductivity in vicinity of**

**Mukwonago Basin.** Note: the blue boundary outlines the Mukwonago Basin.


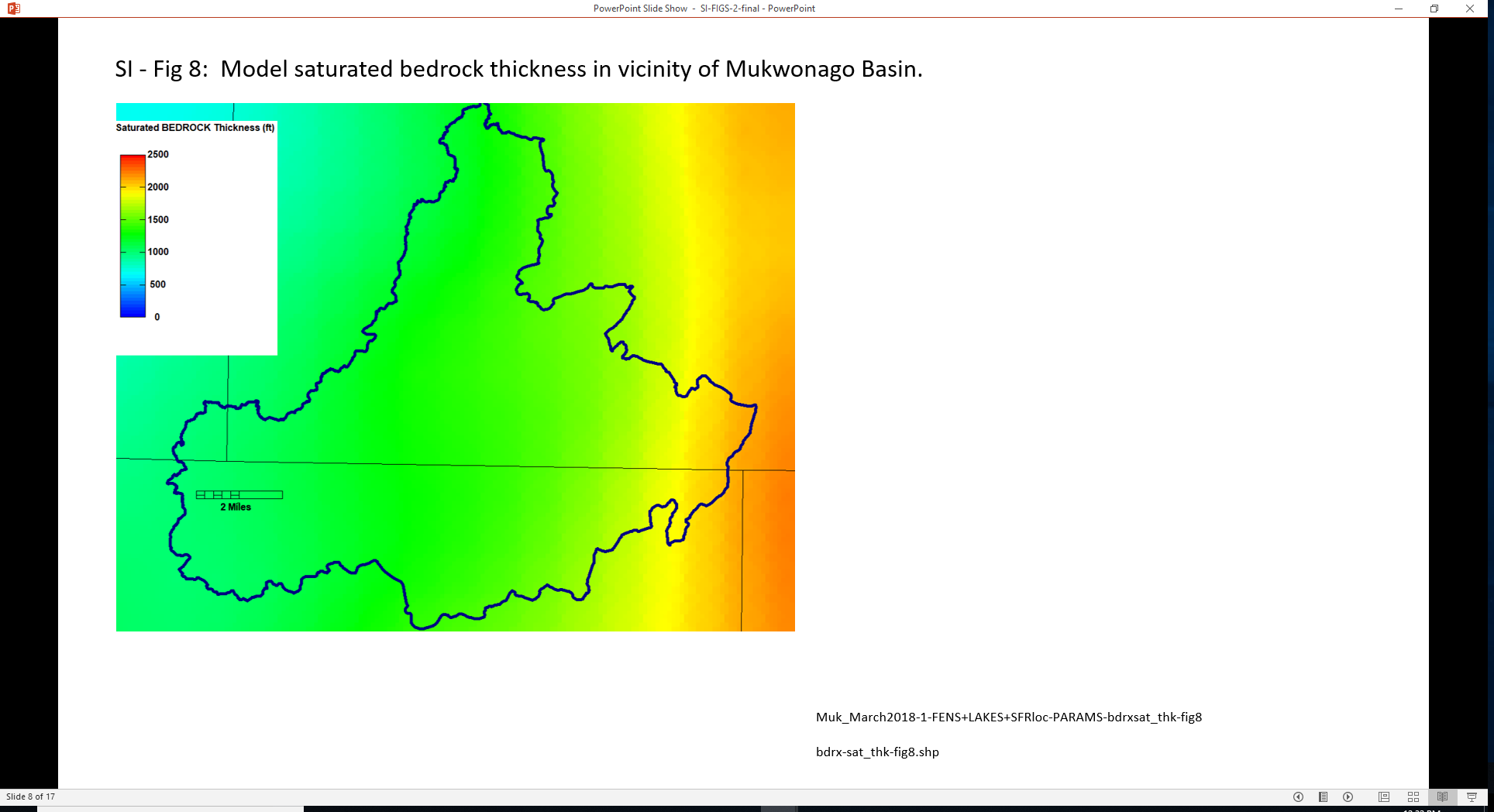


**Figure S8. Model saturated bedrock thickness in vicinity of Mukwonago Basin.** Note: the blue boundary outlines the Mukwonago Basin.


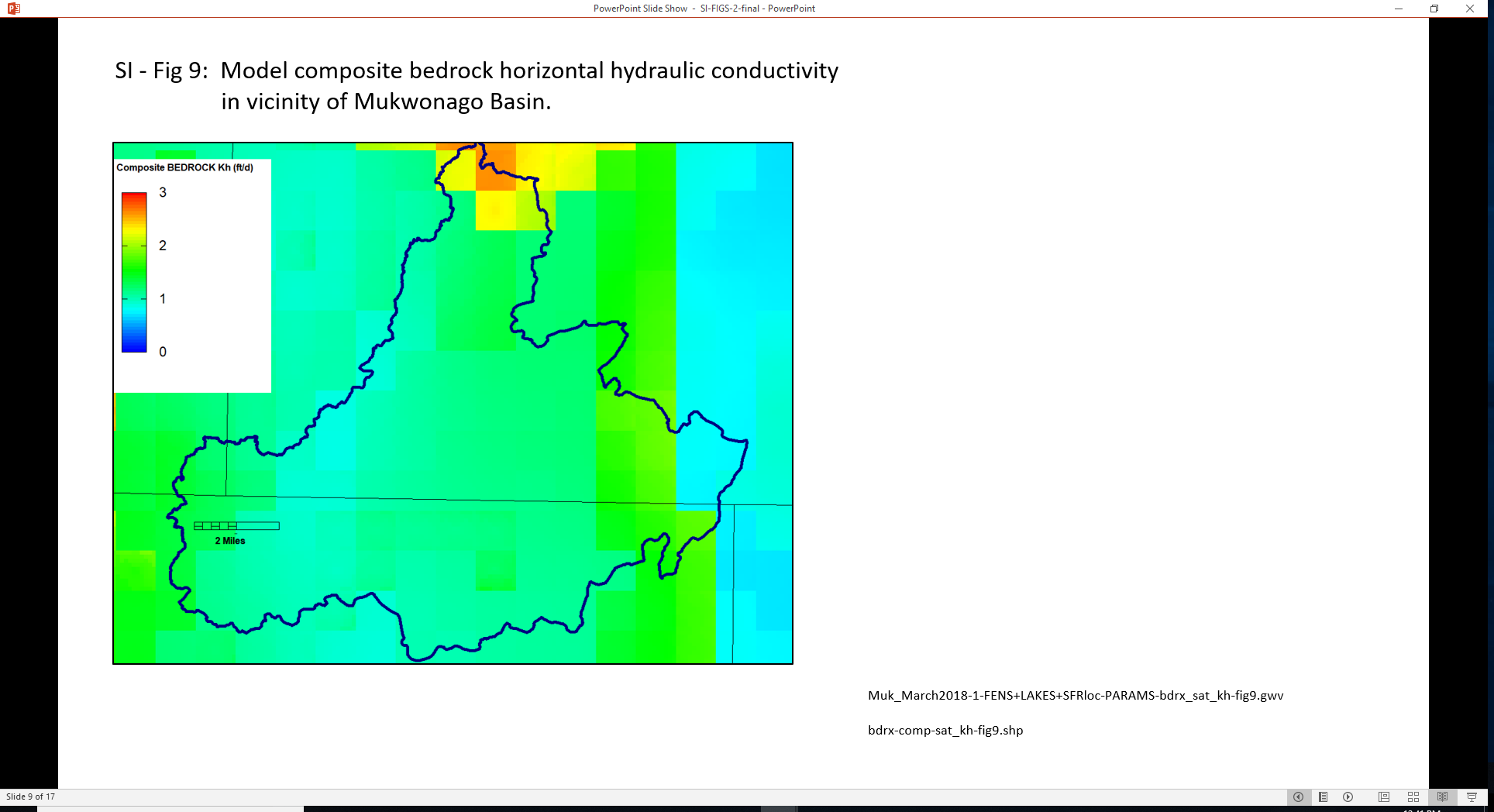


**Figure S9. Model composite bedrock horizontal hydraulic conductivity in vicinity of**

**Mukwonago Basin**. Note: the blue boundary outlines the Mukwonago Basin.

7. Aquifer stresses.

The stresses active in the MGWF model are infiltration across the root zone and pumping from glacial and bedrock wells. Both sets of inputs are inherited directly from the parent UPFOX model.

The UPFOX model adds recharge directly to the water table by means of the MODFLOW recharge (RCH) package (Feinstein et al., 2010; Feinstein et al., 2018). The steady-state recharge rates were estimated over 5000 ft by 5000 ft (1524 m by 1524 m) blocks using the Soil-Water Balance (SWB) Method as the average yearly quantity of water that passed through the root zone in the year 2000 after losses to runoff and evapotranspiration (Westenbroek et al., 2009). In the MGWF model the Unsaturated Zone (UZF) package (Niswonger et al., 2006; Niswonger et al., 2009) replaces the recharge package and the SWB-generated steady-state rates are input through an infiltration rather than a recharge array (Fig. S10). As emphasized in the published article, the use of the UZF package means that not all infiltration becomes recharge to the water table: a part is rejected when the simulated water table is close to the land surface (for more discussion see Section 8 below).

The pumping input to the MGWF model reflects 2005 estimates (that is, wells with estimated pumping that in periods is greater than 70 gallons per minute (0.265 m^3^ per minute)) for high-capacity wells originally input to the LMB and UPFOX models (Feinstein et al., 2010; Feinstein et al., 2018). These wells are represented in MODFLOW by the Multiple Well Node (MNW2) package. In the vicinity of the Mukwonago Basin (Fig. S11), the total glacial pumping from 10 high-capacity wells is 0.07 million gallons per day (268 m^3^/day) and the total bedrock pumping from 7 high-capacity wells is 0.67 million gallons per day (2544 m^3^/day). These pumping rates, especially for the shallow glacial wells, are low in comparison with surrounding basins in southwestern Wisconsin, implying that the groundwater system is closer to its natural state than in nearby areas more heavily stressed by pumping.


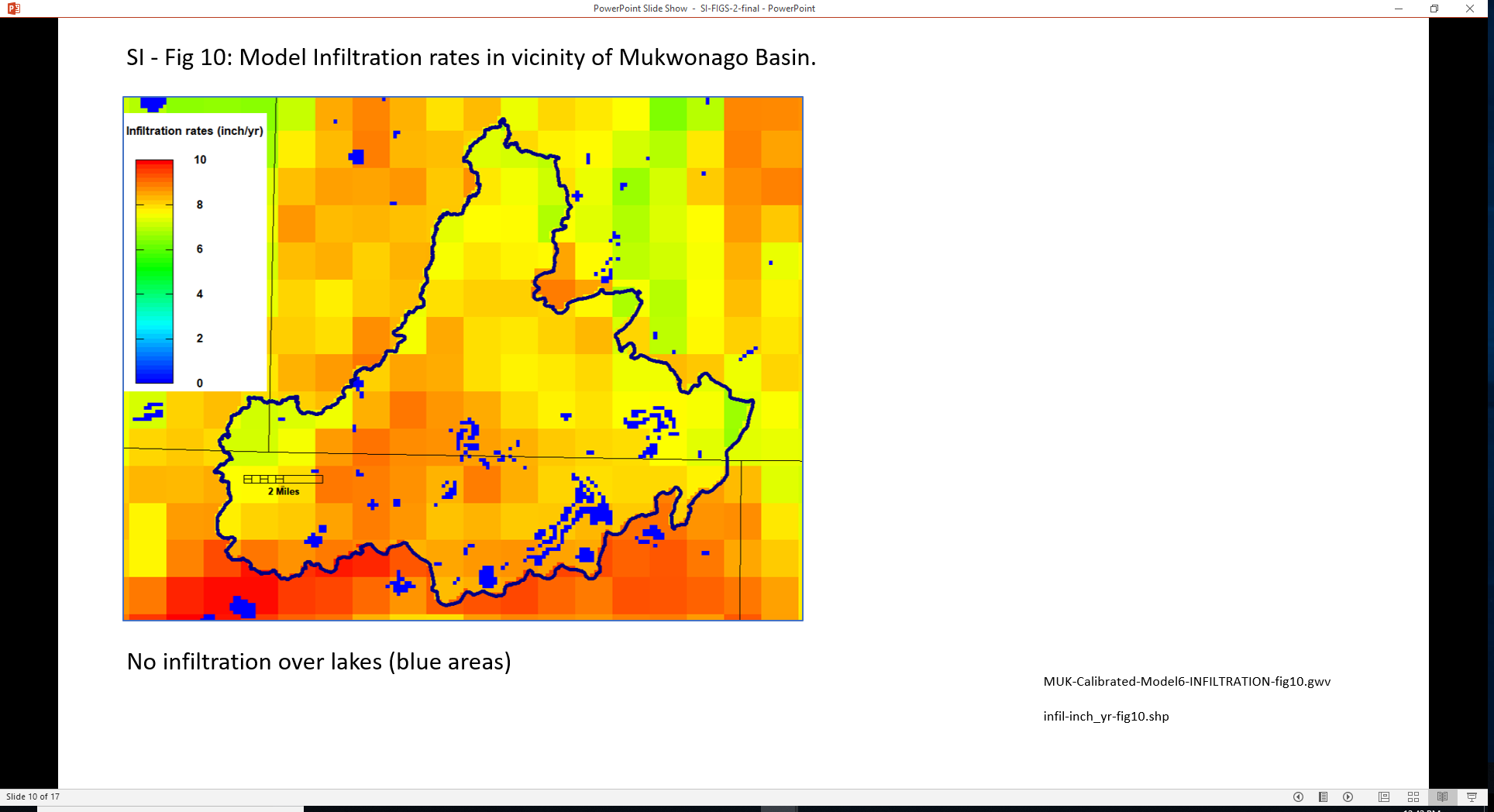


**Figure S10. Model infiltration rates estimated by SWB in vicinity of Mukwonago Basin.** Note: The blue areas are water bodies where infiltration is set to zero. The blue boundary outlines the Mukwonago Basin.


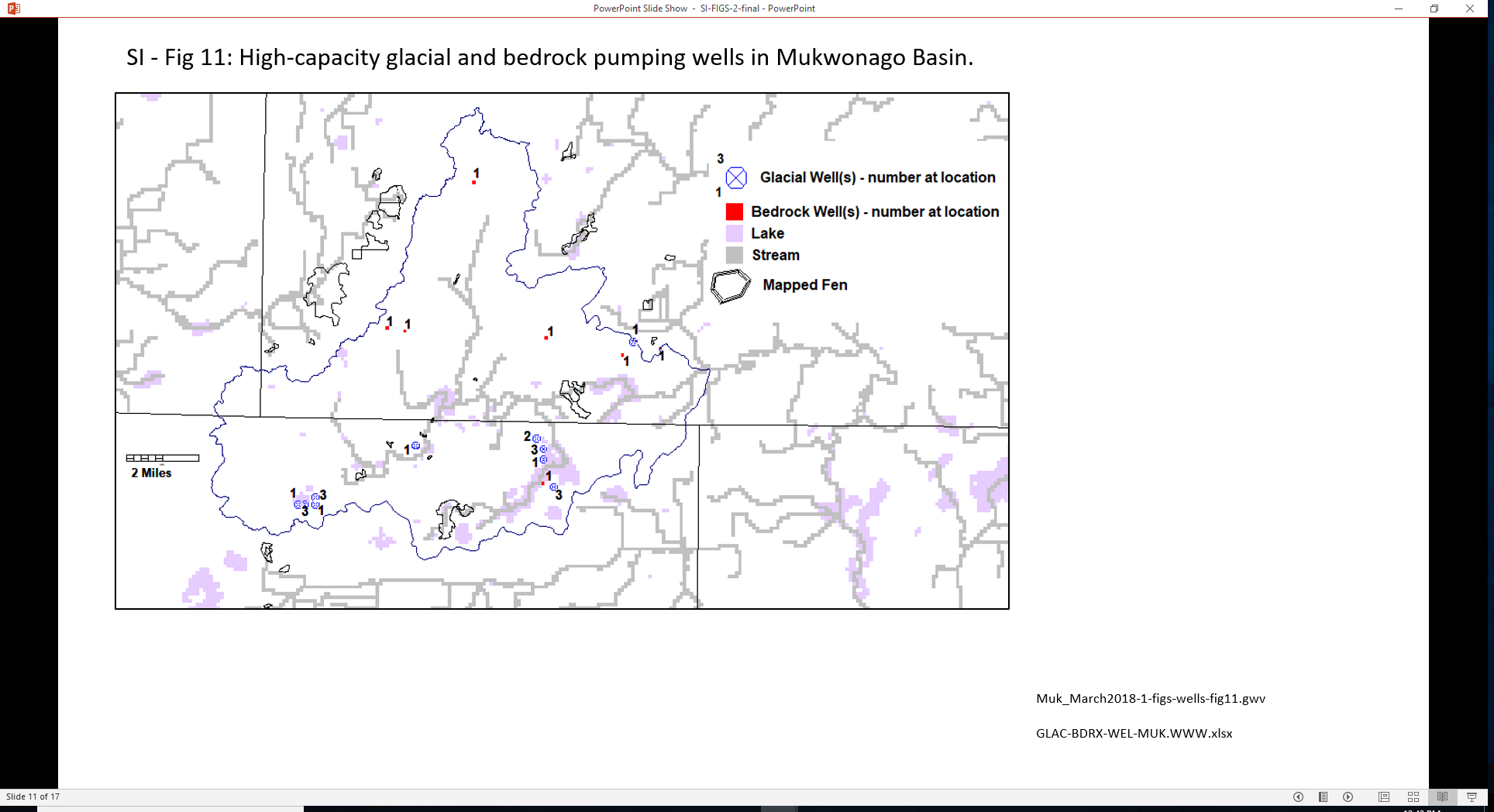


**Figure S11. High-capacity glacial and bedrock pumping wells in Mukwonago Basin.** Note: the blue boundary outlines the Mukwonago Basin. Mapped fens from Wisconsin Department of Natural Resources wetlands inventory (2011).

8. Simulation of seepage to land surface.

The UZF package not only simulates recharge and rejected recharge, but also seepage, that is leakage to the land surface. The published article contains a discussion of the formulation for calculating the seepage flux (in units of volume per time) and its dependence on the simulated water-table elevation, the land surface input as the top of layer 1 at each row/column location, the vertical hydraulic conductivity of the water-table layer, and the parameter SURFDEP, corresponding to the undulation amplitude expected for the land surface in lowland areas. With the UZF package, MODFLOW-NWT is able to simulate seepage with the following input:

- A header line containing the value for SURFDEP - set to two feet in the MGWF model on the basis of the standard deviation of the excursion of the land surface elevation in lowland basin areas identified with mapped wetlands containing persistent standing water or wet soils over at least one quarter of the cell area (Wisconsin Department of Natural Resources, accessed November 2011 at [*https://dnr.wi.gov/topic/wetlands/inventory.html*](https://dnr.wi.gov/topic/wetlands/inventory.html).)
- A cell-by-cell array called IUZFBND, which indicates where infiltration is applied to the water table layer and UZF is active,
- A cell-by-cell array called IRUNBND, which indicates to the SFR segment number where water derived from any seepage or rejected recharge at the cell is instantaneously routed,
- A cell-by-cell array called FINF, which contains the infiltration rates (in length per time units) that cross the root zone, as estimated by SWB (shown in Fig. S10).

For the first set of sensitivity runs discussed in the main article, the value of SURDEP in the header line was modified. For the second sensitivity run, an additional array of vertical hydraulic conductivity values was inserted after the IRUNBND array to change the K_v_ values applied to the seepage and rejected recharge calculations from the default values associated with layer 1 of the model.

No unsaturated parameters (such as an array of unsaturated K_v_ or a value for saturated porosity, residual water content or wilting point, or a Brooks-Corey exponent governing the characteristic curve relating unsaturated K_v_ to water content) are needed to simulate recharge, rejected recharge, and seepage under steady-state conditions. Moreover, the IRUNBND array is only needed if simulated baseflow is expected to include the simulated groundwater runoff for rejected recharge and seepage, for example, when performing a flux calibration (as in the current application, see Section 9 below).

For the MGWF model through the IUZFBND array, UZF is set inactive over lake (DRN) cells, as indicated by dark blue areas in Figure S10 for the Mukwonago Basin. The lakes are assumed to occupy the entire cell area, ruling out the possibility of recharge to the water table or seepage to the land surface. However, UZF is set to active in cells containing stream channels because recharge and riparian seepage can occur within the 500 ft by 500 ft (152.4 m by 152.4 m) area occupied by a cell hosting a SFR boundary condition. In fact, almost two-thirds of the seepage simulated for the Mukwonago Basin occurs in SFR cells (see published article).

9. Calibration process and summary of output.

The MGWF model was calibrated to sets of head and baseflow targets specially developed for its domain, with emphasis on the Mukwonago Basin (the model nearfield). The target list consists of 11 nearfield baseflow targets in upland areas with low flows, 5 nearfield baseflow targets in more lowland areas with larger flows, 1076 farfield head targets from shallow (glacial and upper bedrock) wells, and 1009 nearfield head targets from shallow wells. The baseflow targets incorporate not only the steady-state groundwater discharge to SFR cells upgradient of the target stream location, but also the discharge to DRN cells representing lakes in connection with the upgradient stream network.

The head targets (Fig. S12) were derived from well construction reports (WCRs) reported to the Wisconsin Department of Natural Resources (Wisconsin Department of Natural Resources website, accessed June 2016 at <https://dnr.wi.gov/topic/Groundwater/data.html>). The head target value was calculated by subtracting the depth to water recorded in the WCR from the land surface elevation. The land surface elevation was determined by intersecting the well elevation with county LIDAR elevation data in ArcGIS. For this reason, those WCRs that were located using GPS or had been manually moved to the home or driveway of the address listed on the WCR giving a location accuracy of better than 150 feet were used. Wells with poor locational accuracy have greater error in their land surface elevations and were not used. Less than 5 percent of these head targets were removed from the calibration set because they were screened in the glacial material but intersected bedrock in the model or vice versa. This occasionally happens when during model construction where the bedrock surface varies more than can be reasonably represented in the model grid. The remaining head targets were imported into the model for calibration (Feinstein et al., 2019.)

The flux targets (Fig. S12) were derived from stream flow measurements made by Gittings (2005) and those made for this study using the current meter mid-section method. We used a Marsh-McBirney EM meter and the sixth-tenths depth method to measure mean stream velocities in each cross section of the streams (Turnipseed and Sauer, 2010). The measurement included at least 20 measurement sections per stream or at a minimum of0.2-ft intervals if the stream was narrow, generally less than 4 feet across.

At the four sites with more than eight measurements, we compared the measured flows to the flows at the Mukwonago River where it discharges into the Fox River at the very downstream part of the basin, USGS Station: 05544200 (https://waterdata.usgs.gov/usa/nwis/uv?05544200). A power law relationship was derived between the two flows (Gebert et al., 2007). A baseflow estimate of the Mukwonago at the Fox was determined using the HySEP local minimum hydrograph separation tool in the USGS Groundwater Toolbox (Kiang et al., 2018). HySEP local minimum gave a baseflow estimate of 39 cubic feet per second for the period from 1973 to 2017. This average value was assumed to apply under steady-state conditions. It is intermediate those from the BFI Standard and HySEP fixed intervals tools. Using the power law relationship and the baseflow estimate at the outlet, the baseflow for the four sites was determined. These flows and the flow at the Mukwonago River at the outlet of the Basin are shown in the upper right of Figure S13.

At the other stream flow sites, the flows are generally much less and with fewer measurements. The ratio between flows in the Mukwonago River outlet at the Fox River and the flows at the other sites on the day of their measurements was used to calculate the flow at these sites corresponding to baseflow. These estimated flows are shown in the upper left of Figure S13 and are the unweighted baseflow targets.


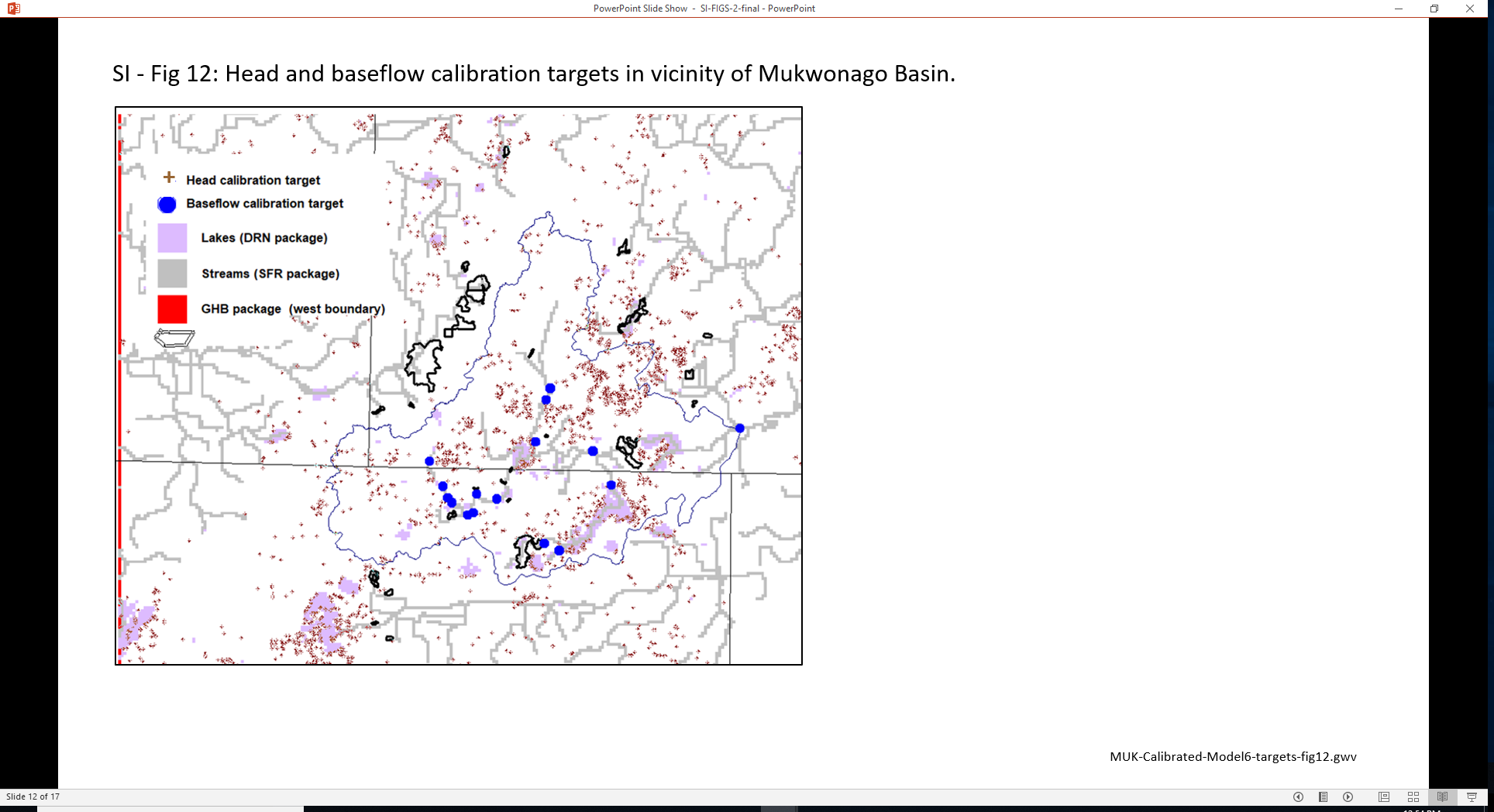
**Figure S12. Head and baseflow calibration targets in vicinity of Mukwonago Basin.** Note: the blue boundary outlines the Mukwonago Basin.

Model simulations were performed with the Newton-Raphson solver (Niswonger et al., 2011) with tight criteria for convergence (flux tolerance set to 3 ft^3^/day = 0.08 m^3^/day). The calibration process was performed by nonlinear regression using the PEST program (Doherty, 2008a, Doherty, 2008b), applying the Singular Value Decomposition method across five iterations (at which point the improvement to the agreement for observed and simulated values at targets levelled off). Targets were weighted in order to give roughly equal power in estimating parameters to head and baseflow targets. Scatter plots of the final fit to the observed sets of baseflow and head targets are shown in Figure S13.

A third type of target is computed by penalizing false positives for seepage, that is, by summing the number of cells where simulated seepage is greater than 0.025 ft^3^/day (0.00071 m^3^/day) in areas in the Mukwonago Basin not coincident with the mapped fens shown in figure S4. Whereas the calibration process starting with initial parameter values did improve the simulated fit to observed heads and baseflow targets, there was negligible improvement in the fit to reproducing fen areas from that achieved with the initial set of parameter values.

The following table lists the model parameters estimated by the PEST calibration process. The initial values for parameters were derived from the parent UPFOX model. Some parameters were updated in terms of a single value, some in terms of multipliers applied to one or more arrays of values:

Parameter Update at end of calibration process___

Streambed K_v_ Value updated from 5 to 4.99947 ft/day

Lakebed conductance Multiplier on initial values = 2.72347

Kh of glacial layer 1 Multiplier on initial values = 1.35925

Kh of glacial layer 2 Multiplier on initial values = 0.605640

Kh of glacial layer 3 Multiplier on initial values = 1.05079

Kv of glacial layer 1 Multiplier on initial values = 0.821801

Kv of glacial layer 2 Multiplier on initial values = 1.06649

Kv of glacial layer 3 Multiplier on initial values = 1.00823

Infiltration Multiplier on initial values = 1.07365

The most important updates are the increase in the Kh of layer 1 (by a factor of 1.36) and the increase in infiltration (by a factor of 1.07). While these two changes tend to counteract each other in terms of the elevation of simulated heads, they act in concert to maintain seepage to the six mapped fen areas by concentrating flow in the shallowest part of the flow system.


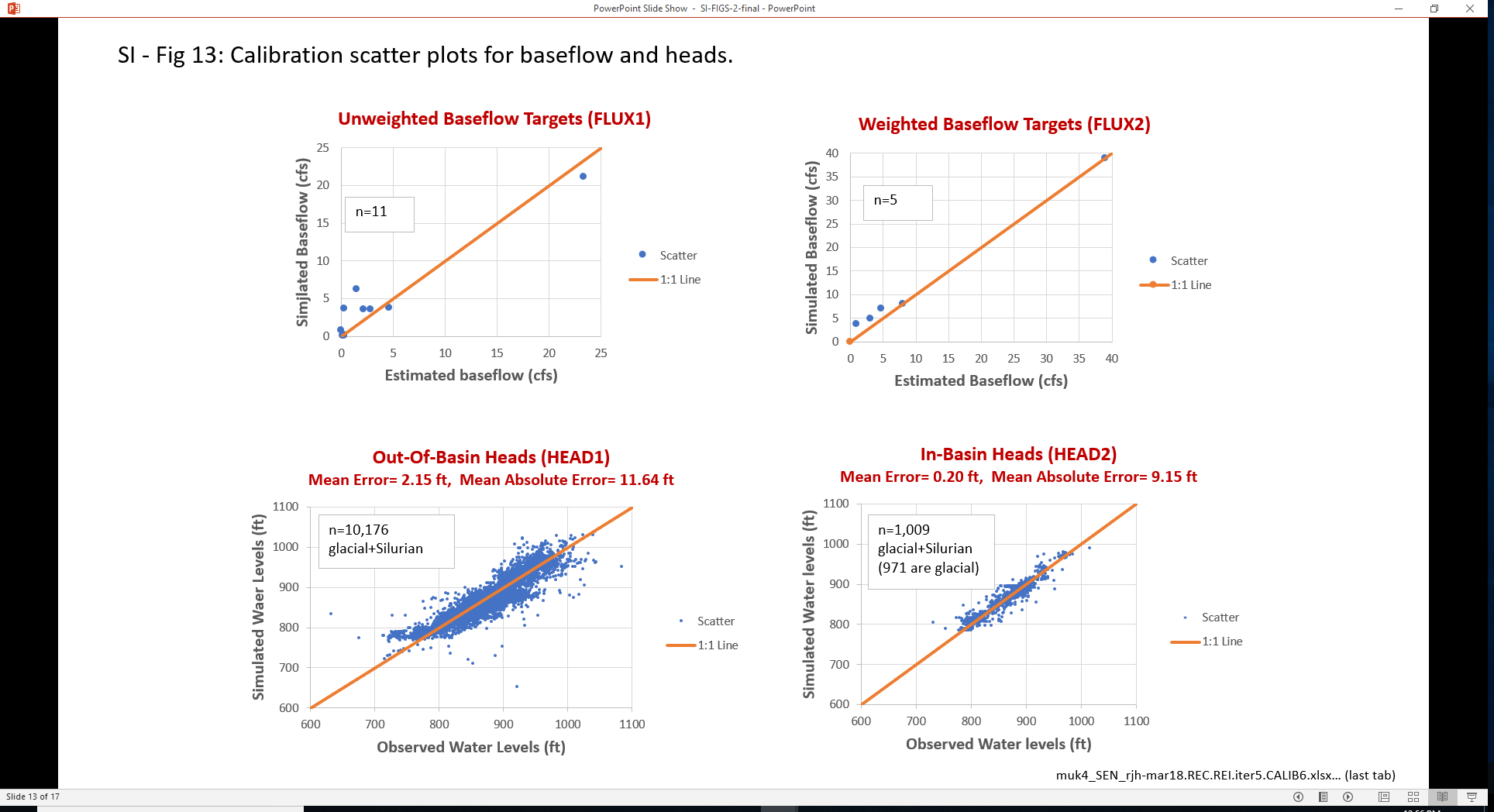


**Figure S13. Calibration scatter plots for stream baseflow and groundwater heads.**

The output of the calibrated MGWF model can be summarized in terms of the nearfield water table elevation (Fig. S14) and the water budget for the Mukwonago Basin (Fig. S15). The simulated water table reflects the flow from upland areas to the valleys occupied by surface-water bodies. The simulated water budget, incorporating flow through all glacial and bedrock layers, reports the groundwater exchange with neighboring basins, the nearfield sources of water (in terms of recharge to the water table as well and loss from stream baseflow to groundwater), and the nearfield sinks of water (in terms of seepage to the land surface, discharge to lakes, discharge to stream channels, and withdrawals from pumping wells). It is notable that the nearfield sources to groundwater discharge roughly equally to seeps, lakes and stream channels. However, as pointed out earlier, much of the seepage occurs within SFR cells, that is within riparian corridors immediately adjacent to stream channels. The seepage outside of SFR cells amounts to about 37% of total seepage, and about two-thirds of this flux is concentrated at mapped fen locations.


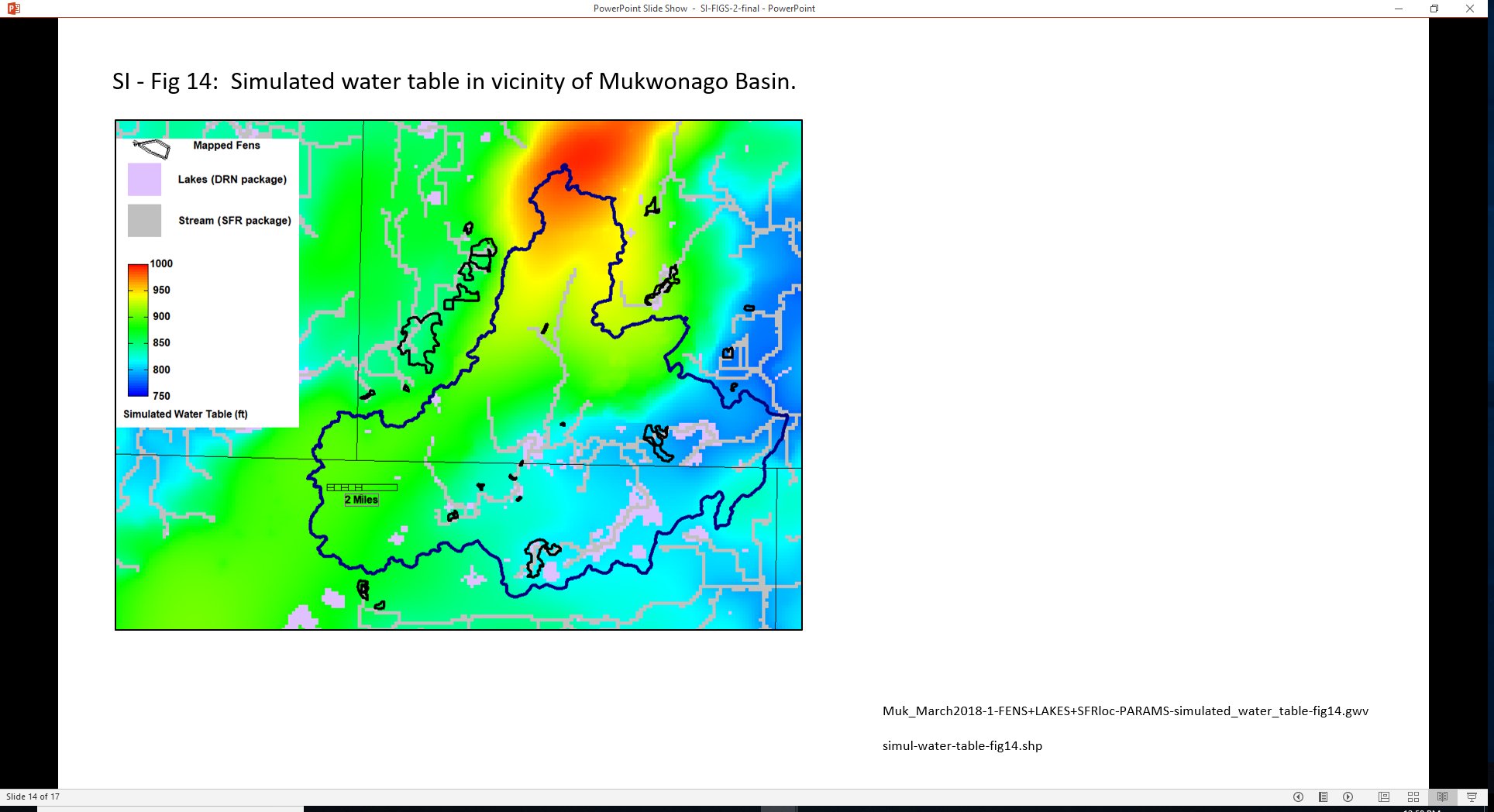


**Figure S14. Simulated water table in vicinity of Mukwonago Basin.** Note: the blue boundary outlines the Mukwonago Basin.


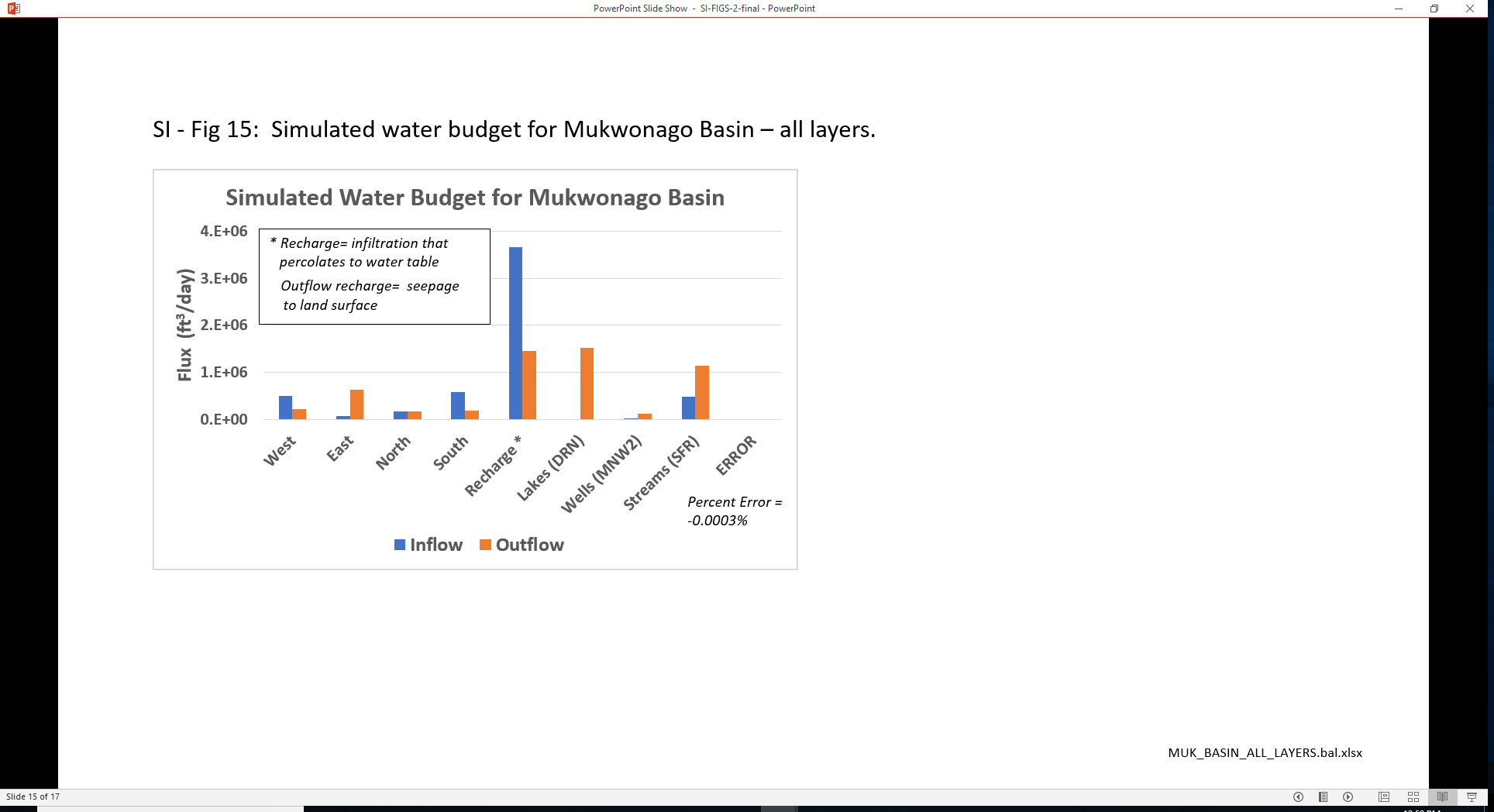


Figure S15. Simulated water budget for Mukwonago Basin – all layers.

10. Model limitations.

The MGWF model shares many of the limitations associated with the parent UPFOX model, including those attributable to the glacial layering and the lateral cell dimensions (see discussion in Feinstein et al., 2018). However, particular limitations arise from the central objectives of the MGWF model as described in the published article: to evaluate fen discharge and the vulnerability of fens to shallow pumping. These limitations, discussed more fully in the published article, fall into several categories:

- The neglect of unsaturated flow conditions;
- The neglect of transient flow conditions in the unsaturated as well as the saturated zones;
- The implications of the lateral cell size (equivalent to about 5.7cres or 2.3 hectares) for any attempt to simulate small fens associated with subtle changes in topography;
- The need to correlate the threshold for fen discharge to the vegetation supported by the fens.

11. Acknowledgments

We thank The Nature Conservancy for funding this work as part of its ongoing efforts to protect water resources. The U.S. Geological Survey also provided support through the USGS Cooperative Matching Funds program. The authors acknowledge and thank Andrew Leaf and Paul Juckem for working out many mechanical aspects of the method as part of their U.S. Geological Survey studies of basins in northern Wisconsin. We also thank Megan Haserodt and Keith Halford for insightful review comments.

Any use of trade, firm, or product names is for descriptive purposes only and does not imply endorsement by the U.S. Government.

12. References.

Doherty, John, 2008a, PEST, Model Independent Parameter Estimation—User manual (5th ed.):

Brisbane, Australia, Watermark Numerical Computing, accessed October 1, 2009, at [*http://www.pesthomepage.org/Downloads.php*](http://www.pesthomepage.org/Downloads.php)*.*

Doherty, John, 2008b, PEST, Model Independent Parameter Estimation—Addendum to user

manual (5th ed.): Brisbane, Australia, Watermark Numerical Computing, accessed October 1, 2009, at [*http://www.pesthomepage.org/Downloads.php*](http://www.pesthomepage.org/Downloads.php)*.*

Feinstein, D.T., R.J. Hunt, and H.W. Reeves. 2010, Regional groundwater-flow model of the

Lake Michigan Basin in support of Great Lakes Basin water availability and use studies.

*U.S. Geological Survey Scientific Investigations Report 2010–5109*, 379 p.

Feinstein, D.T., M.N. Fienen, J.L. Kennedy, C.A. Buchwald, and M.M. Greenwood. 2012,

Development and application of a groundwater/surface-water flow model using MODFLOW-NWT for the Upper Fox River Basin, Southeastern Wisconsin*. U.S. Geological Survey Scientific Investigations Report 2012–5108*, 124 p.

Feinstein, D.T., L.J. Kauffman, M.J. Haserodt, B.R. Clark, and P.F. Juckem. 2018. Extraction

and development of inset models in support of groundwater age calculations for glacial aquifers. *U.S. Geological Survey Scientific Investigations Report 2018-5038*, 96 p. DOI: 10.3133/sir20185038.

Feinstein, D.T., D.J. Hart, S. Gatzke, R.J. Hunt, R.G. Niswonger, and M.N. Fienen. 2019.

A Simple Method for Simulating Groundwater Interactions with Fens to Forecast Development Effects. *Journal of Groundwater.*

Feinstein, D.T., R.J. Hunt, and H.W. Reeves, H.W., 2010: Regional groundwater-flow model of

the Lake Michigan Basin in support of Great Lakes water availability and use studies. U.S. Geological Survey Scientific Investigation Report 2010-5109, 379 p.

Gebert, W.A., J.F. Walker, and J.L. Kennedy. 2011. Estimating 1970-99 average annual

groundwater recharge in Wisconsin using streamflow data. U.S. Geological Survey Open-File Report 2009-1210, 14 p. plus appendixes.

Gittings, H.E., 2005. Hydrogeologic controls on springs in the Mukwonago River watershed, SE
 Wisconsin. M.S. thesis*, University of Wisconsin-Madison, Department of Geology and*

*Geophysics*. 141 p.

Haserodt, M.J., L.J. Kauffman, and D.T. Feinstein. 2018. MODFLOW-NWT inset models from

the regional Lake Michigan Basin Model in support of groundwater age calculations for glacial aquifers. U.S. Geological Survey data release: https//doi.org/10.5066/F76D5R5V

Jones, P.M. and D.T. Feinstein. 2019. MODFLOW inset model for the Mukwonago Basin

with implementation of the UZF package to simulate fen seepage. *U.S. Geological Survey data release.* https://doi.org/10.5066/P9UUB30I.

Kiang, J.E., K.M. Flynn, Tong Zhai, Paul Hummel, and Gregory Granato. 2018, SWToolbox: A

surface-water toolbox for statistical analysis of streamflow time series: *U.S. Geological Survey Techniques and Methods*, book 4, chap. A–11, 33 p.

Niswonger, R.G., and Prudic, D.E., 2005, Documentation of the Streamflow-Routing (SFR2)

Package to include unsaturated flow beneath streams—A modification to SFR1. *U.S. Geological Survey Techniques and Methods*, book 6, chap. A13, 50 p.

Niswonger, R.G., D.E. Prudic, and R.S. Regan. 2006. Documentation of the unsaturated-zone

Flow (UZF1) package for modeling unsaturated flow between the land surface and the water table with MODFLOW-2005. *U.S. Geological Survey Techniques and Methods*, book 6, chap. A19: 62 p.

Niswonger, R.G. 2009. Changes to the UZF1 package for release version 1.7:

4 p., accessed April 2, 2019 at [*https://water.usgs.gov/ogw/modflow/MODFLOW-2005-Guide/readme_UZF.pdf*,](https://water.usgs.gov/ogw/modflow/MODFLOW-2005-Guide/readme_UZF.pdf,%20%20%20%20%20%20%20%20%20%20%20%20%20%20%20%20%20%20%20%20%20%20%20%20%20%20)

Niswonger, R.G., Panday, S., and Ibaraki, M., 2011, MODFLOW- NWT, A Newton

formulation for MODFLOW-2005*. U.S. Geological Survey Techniques and Methods* 6-A37, 44 p.

Turnipseed, D.P., and V. B. Sauer, 2010, Discharge measurements at gaging stations. *U.S. Geological Survey Techniques and Methods* book 3, chap. A8, 87 p.

U.S. Geological Survey, 2014, National Elevation Dataset (NED) 1/3 arc-second

downloadable data collection, accessed May 28, 2015, at [*https://www.sciencebase.gov/catalog/item/4f70aa9fe4b058caae3f8de5*](https://www.sciencebase.gov/catalog/item/4f70aa9fe4b058caae3f8de5).

Westenbroek, S.M., Kelson, V.A., Dripps, W.R., Hunt, R.J., and Bradbury, K.R., 2009, SWB—

A modified Thornthwaite-Mather soil-water balance code for estimating ground-water recharge. *U.S. Geological Survey Techniques and Methods* 6–A31, 65 p.

Wisconsin Department of Natural Resources website, accessed 22 April 2019 at

[*https://dnr.wi.gov/water/watershedsearch.aspx*](https://dnr.wi.gov/water/watershedsearch.aspx).

Wisconsin Department of Natural Resources website, accessed November 2011 at

[*https://dnr.wi.gov/topic/wetlands/inventory.html*](https://dnr.wi.gov/topic/wetlands/inventory.html)*.*

Wisconsin Department of Natural Resources website, accessed June 2016 at

<https://dnr.wi.gov/topic/Groundwater/data.html>
